# UbC4 from Leishmania is a Druggable E2 Ubiquitin Conjugating Enzyme: Structural Basis and Fragment Hits for Future E2-Recruiting PROTAC Development

**DOI:** 10.1021/acsomega.6c05158

**Published:** 2026-09-08

**Authors:** Cécile Exertier, Lorenzo Antonelli, Anastasia Liuzzi, Marcus Ruffa, Vittorio Brufani, Gianni Colotti, Annarita Fiorillo, Andrea Ilari

**Affiliations:** † Institute of Molecular Biology and Pathology, Italian National Research Council (IBPM-CNR), c/o Department of Biochemical Sciences, Sapienza University of Rome, Ed. CU027, P.le A. Moro 5, 00185 Rome, Italy; ‡ Department of Biochemical Sciences, Sapienza University of Rome, Ed. CU027, P.le A. Moro 5, 00185 Rome, Italy

## Abstract

Leishmaniasis is a neglected disease that affects around two million people every year. Current treatments are often highly toxic or prone to resistance, underscoring the urgent need for new therapeutic strategies. PROTACs may offer a promising alternative, as they can potentially mitigate both toxicity and resistance. However, very little is known about the ubiquitin–proteasome system (UPS) in *Leishmania*. Notably, only two E3 ligases containing a CULT domain have been identified so far, and none carrying a von Hippel–Lindau (VHL) domain—the classical E3 ligase used by clinically advanced PROTACs. In this work, we take an important step toward understanding the UPS in *Leishmania*, and we propose that in this organism the UbC4 E2 enzyme, rather than an E3, may be directly exploited to develop a PROTAC able to engage a protein of interest. Here, we report the biochemical and structural characterization of the *Leishmania major* ubiquitin-conjugating enzyme 4 (UbC4). Through a fragment screening campaign, we identified 10 fragments binding to distinct cavities on UbC4. Among these, five interact with the same noncatalytic pocket that is poorly conserved in humans, while one fragment binds near the catalytic cysteine. Using DeepFrag predictions and molecular docking, we explored fragment elongation strategies to enhance affinity for their respective binding sites, with the goal of guiding the development of E2-recruiting PROTACs or UPS inhibitors for the treatment of leishmaniasis.

## Graphical abstract

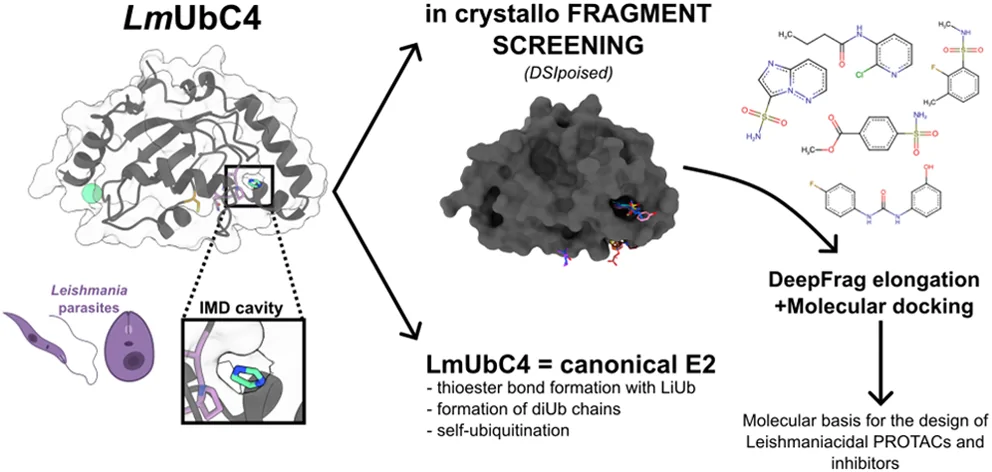

## Introduction

An estimated 700 thousands to 1 million new cases of leishmaniasis are reported by the World Health Organization each year (www.who.int). Leishmaniases belong to the class of neglected tropical diseases caused by protozoan parasites of the genus *Leishmania*, comprising over 20 different species associated with distinct disease phenotypes. *Leishmania* parasites are transmitted to the human host during the blood meal of female phlebotomine sandflies. While their primary habitat includes tropical and subtropical regions, their range is increasingly expanding toward more temperate regions.

Leishmaniasis manifests in three primary clinical forms: (i) cutaneous leishmaniasis (CL), characterized by skin lesions leaving life-long scars; (ii) mucocutaneous leishmaniasis (MCL), which causes the partial or total destruction of mucous membranes of the nose, mouth, and throat; and (iii) visceral leishmaniasis (VL), also known as kala-azar, the most severe form, whose symptoms include fever, anemia, and enlargement of internal organs (splenomegaly and hepatomegaly). If left untreated, VL is frequently fatal.^1–4^

Current chemotherapeutic options for leishmaniasis include amphotericin B, antimonials (*e.g*., meglumine antimonate), paromomycin, and pentamidine, as well as newer agents such as miltefosine and sitamaquine. However, these treatments remain suboptimal due to limited efficacy, adverse side effects, and the increasing emergence of drug-resistant parasite strains.^5,6^ Moreover, the precise mechanisms of action of several of these compounds are still not fully elucidated.^7–11^

Recently, efforts have been put into the development of novel therapeutic solutions, such as nanovaccines, endochin-like quinolones that affect the electron transport chain, benzophenone-derived bisphosphonium salts that target complex II of the parasite respiratory chain, and tafenoquine that leads to mitochondrial dysfunction; however, none of these solutions have reached the market to date.^6,12^

Targeting the ubiquitin–proteasome system (UPS) has emerged as a promising therapeutic strategy against cancer and autoimmune diseases, with recent proposals suggesting its utility in tackling infectious diseases, including parasitic infections.^13,14^ Ubiquitination is a post-translational modification known to target proteins toward proteasomal degradation; it also regulates DNA damage repair and cell cycle progression, and more. In Trypanosomatids, proteolysis is crucial for regulating various cellular processes throughout the parasite’s life cycle,^15^ especially in carefully controlling the morphological changes during differentiation. This underscores the vital role of the UPS in *Leishmania*.

The UPS utilizes a cascade of enzymes to covalently attach ubiquitin (Ub) to the lysine residues of a protein substrate. Ubiquitin itself contains seven lysine residues (K6, K11, K27, K29, K33, K48, and K63) and an N-terminal methionine (M1) that can also be ubiquitinated, enabling the formation of different linear or branched chains of Ub.^16^

However, very little is known about the *Leishmania* UPS yet. Of note, genomic analysis of *Leishmania mexicana* has identified 2 ubiquitin-activating enzymes (E1s/UbAs), 13 ubiquitin-conjugating enzymes (E2s/UbCs), 79 ubiquitin ligases (E3s) including 12 HECT E3s, 1 RBR E3, 5 U-box RING E3s, 57 RING and 4 RING-CH-type E3s, and 20 deubiquitinating cysteine peptidases (DUBs).^17^

According to the state-of-the-art knowledge about the human UPS, the E1 activates ubiquitin (Ub) through an ATP-dependent adenylation of its C-terminal carboxyl group, enabling the formation of a thioester bond between the E1 and Ub. Ub is then transferred to the catalytic cysteine of an E2 through a trans-thioesterification. From there, HECT E3 ligases form a thioester intermediate with Ub before the ubiquitination of the substrate, whereas RING E3 ligases promote the direct transfer of Ub from an E2-Ub to the ε-amino group of a substrate lysine (Figure S1).^18^ It is worth mentioning that, in the human genome, 600 genes encoding for RING E3 ligases (57 in *L. mexicana* ^17^) against 30 gene of HECT E3 ligases (12 in *L. mexicana* ^17^) were identified, suggesting that the most common ubiquitination scheme might concern Ub direct transfer from the E2 to the substrate, the RING E3 ligase serving more as an intermediate that bridges the E2 and the protein substrate together.^19^ The ubiquitination is repeated several times to polyubiquitinate the substrate, yielding a signal for degradation by the proteasome.

Previous studies have shed light on the importance of the parasite proteasome and of the DUBs in the *Leishmania* life cycle; recently, GNF6702 and LXE408 proteasome inhibitors have shown promising efficiency on *Leishmania*.^20,21^ Previous investigations also underlined the key role of E1s, E2s, and E3s in the differentiation of *Leishmania*: among others, *ubc1*/*cdc34*, *ubc2*, *uev1*, and *hect2* genes appear essential for the transformation from the promastigote to the amastigote stage.^17,22^

Recently, PROTACs (proteolysis targeting chimeras) have emerged as a potential leishmanicidal strategy.^13^ PROTACs are heterobifunctional compounds recruiting E3 ligases and binding a chosen target protein. PROTACs allow the forced ubiquitination and subsequent degradation of the target via the UPS. Notably, several PROTAC candidates targeting proteins involved mainly in cancers, such as breast cancers and lymphomas, are currently being evaluated in clinical trials.^23^ Although most PROTACs recruit E3 ligases, they can also be designed to recruit E2s directly, and some evidence already proved E2-PROTACs efficiency.^24,25^

However, the current lack of structural data on the UPS of *Leishmania* makes it difficult to comprehend such a crucial cellular process and slows the rational design of chemotherapeutics specifically targeting ubiquitination in *Leishmania*. In this work, we recombinantly produced LiUbA1a (E1), LmUbC4 (E2), and LiUb (ubiquitin). We provide the structural and functional characterization of LmUbC4 (an orthologue of human UbE2H) from *Leishmania major*. Furthermore, we performed a crystallographic fragment screening, a powerful technique previously successful in identifying inhibitors for *Leishmania infantum* trypanothione reductase, leading to the identification of 10 low-affinity ligands for this E2.^26–28^ Finally, we utilized these results in a pipeline combining deep-learning elongation prediction and molecular docking to pave the way toward the design of PROTACs capable of specifically recruiting *Leishmania* E2s to target essential parasite proteins that are otherwise challenging to drug, such as trypanothione reductase (TR).^13^ Indeed, TR is indispensable for the parasite’s survival within the host. However, the discovery of effective inhibitors remains challenging because the enzyme is highly expressed in the amastigote parasite stage and features a large catalytic cavity that is partially exposed to solvent. These characteristics complicate the identification of compounds capable of achieving high-affinity binding and potent inhibition while maintaining favorable drug-like properties.^5^

## Materials and Methods

### Recombinant Expression

#### UbA1a

The gene for UbA1a from *L. infantum* (Uniprot A4I0A5) was synthesized by Optiprime Diagnostic and subcloned into a pGEX4T1 vector. *Escherichia coli* BL21­(DE3) cells, previously transformed, were incubated at 37 °C in LB until the optical density reached 0.9. Overexpression was induced upon the addition of 0.5 mM isopropyl β-d-thiogalactoside (IPTG), and cells were further incubated at 16 °C for 20 h.

#### UbC4

The gene for UbC4 from *L. major* (Uniprot Q4Q5L3), lacking the last C-terminal 67 residues was purchased from Unimed Scientifica and subcloned into a pET14b vector. *E. coli* BL21­(DE3) cells, previously transformed, were incubated at 37 °C in LB until the optical density reached 0.6. Overexpression was induced upon the addition of 1 mM IPTG, and cells were further incubated at 25 °C for 20 h.

#### Ub

The gene for ubiquitin from *L. infantum* (Uniprot E9AHX6) was synthesized by Optiprime Diagnostic and subcloned into a pET28b vector. *E. coli* BL21­(DE3) cells, previously transformed, were incubated at 37 °C in LB until the optical density reached 0.6. Overexpression was induced upon the addition of 0.5 mM IPTG and further incubated at 18 °C for 20 h.

### Purification

#### UbA1

Cells were resuspended in lysis buffer (50 mM Tris pH 8.0, 300 mM NaCl, 5 mM dithiothreitol (DTT), 0.1% Tween20, 5% glycerol, 1 mM ATP, 2.5 mM MgCl_2_) supplemented with one tablet of Pierce cocktail of protease inhibitors (ThermoScientific), 0.1 mM phenylmethylsulfonyl fluoride, and 2 μL of deoxyribonuclease I (Invitrogen). Cells were sonicated twice for 15 min (2 s ON, 10 s OFF), separated for 5 min on ice, and centrifuged to discard cell debris. The soluble fraction was loaded on a previously equilibrated 5 mL GSTrap column (Cytiva). After loading, the column was washed with 5 column volumes (cv) of cleavage buffer (50 mM Tris pH 8.0, 200 mM NaCl, 5 mM CaCl_2_, 1 mM DTT, 5% glycerol). The cleavage of the GST tag was carried out directly on the column using 4 units of thrombin per estimated milligrams of UbA1a bound to the column at 4 °C overnight. The next day, the column was washed with 5 cv of cleavage buffer to recover the tag-free UbA1a. Residual UbA1a-GST and post-cleavage GST were eluted using 50 mM reduced glutathione in lysis buffer. Thrombin was removed upon incubation of the tag-free UbA1a with the benzamidine resin for 30 min on ice. The tag-free protein was then concentrated to be injected on a Superdex 200 Increase 10/300 GL at 0.5 mL/min (running buffer: 20 mM Tris pH 8.0, 150 mM NaCl, 1 mM DTT, 1% glycerol). The peak corresponding to the UbA1a monomer was recovered and concentrated. The tag-free UbA1a monomer was kept at 4 °C overnight and used the next day for the ubiquitination assays.

#### UbC4

Cells were resuspended in lysis buffer 2 (50 mM Tris pH 8.0, 300 mM NaCl, 5 mM β-mercaptoethanol and 5 mM imidazole), supplemented with one tablet of Pierce cocktail of protease inhibitors (ThermoScientific), 0.1 mM phenylmethylsulfonyl fluoride and 2 μL of deoxyribonuclease I (Invitrogen). Cells were sonicated for 40 min (3 s ON, 10 s OFF) and centrifuged to discard cell debris. The soluble fraction was loaded onto a previously equilibrated 5 mL HiTrap column (Cytiva) mounted on a Knauer Azura FPLC instrument. His-tagged UbC4 was eluted using a 5–500 mM imidazole gradient. UbC4 was further purified using size exclusion chromatography (Superdex 200 Increase 10/300 GL) in 20 mM (4-(2-hydroxyethyl)-1-piperazineethanesulfonic acid) (HEPES) pH 7.5, 50 mM NaCl, 1 mM DTT. The protein was concentrated and kept at −80 °C. For ubiquitination assays, UbC4 was cleaved using 4 units of thrombin per mg of protein overnight at 4 °C. Thrombin was removed upon loading of the cleavage mix onto a benzamidine resin for 30 min at 4 °C. Tag-free UbC4 was recovered by reverse immobilized metal affinity chromatography (Protino Ni-TED resin, Macherey-Nagel). The final tag-free UbC4 sample was further concentrated and stored at −80 °C.

#### Ub

Cells were resuspended in lysis buffer 3 (50 mM Tris pH 8.0, 300 mM NaCl, 5 mM β-mercaptoethanol and 5 mM imidazole), supplemented with one tablet of Pierce cocktail of protease inhibitors (ThermoScientific), 0.1 mM phenylmethylsulfonyl fluoride and 2 μL of deoxyribonuclease I (Invitrogen). Cells were sonicated for 40 min (3 s ON, 10 s OFF) and centrifuged to discard cell debris. The soluble fraction was loaded onto a previously equilibrated 5 mL HiTrap column (Cytiva) mounted on a Knauer Azura FPLC instrument. His-tagged Ub was eluted using a 5–500 mM imidazole gradient. Ub was further purified using size exclusion chromatography (Superdex 200 Increase 10/300 GL) in 20 mM Tris pH 8, 100 mM NaCl, 2 mM DTT. The protein was concentrated and kept at −80 °C.

The purity of the protein samples was assessed by sodium dodecyl sulfate-polyacrylamide gel electrophoresis (SDS-PAGE) electrophoresis.

### Ubiquitination Assays

The Ub thioester bond formation with UbC4 was carried out at 37 °C for 15 min to 4 h, and the reaction contained 1.3 μM UbA1a, 10 μM UbC4 and 20 μM Ub. Different purification batches of UbA1a, were used owing to the low purification yield and to ensure reproducibility. Samples were diluted with LDS sample buffer 4× (Genscript) and immediately stored at −20 °C prior to SDS-PAGE electrophoresis. Reaction samples were then loaded onto a 4–12% Bis–Tris SDS-PAGE gel (Genscript) that ran in Tris–MES–SDS running buffer at 200 V (80 mA). The SDS-PAGE gel was stained with Brilliant Blue Coomassie R250. With the first Coomassie-stained SDS-PAGE, a second SDS-PAGE gel ran at the same time in the same exact experimental conditions to proceed with Western blot analysis. Briefly, proteins were transferred to an Immobilon PVDF membrane (Millipore) using a semidry transblot apparatus for 1 h 15 min at 25 V (200 mA). The membrane was blocked with 5% milk in TBS-T buffer for 1 h at 4 °C and then incubated with a 1/1000 dilution of mouse anti-His tag antibody (MCA1396GA, Biorad) overnight at 4 °C. The next day, the membrane was washed and incubated with a 1/5000 dilution of a horseradish peroxidase-conjugated goat anti-mouse IgG antibody (A90-516P Bethyl) for 4 h at 4 °C. Chemiluminescent signal was obtained using the Westar Nova 2.0 kit, and images were collected using a Chemidoc MP imager.

### Crystallization and X-ray Diffraction

H32 crystals of UbC4 (6.5 mg/mL) grew in 3 M NaCl, 0.1 M MES/imidazole pH 6.5, and 20% glycerol using the sitting drop vapor diffusion method. Crystallization plates were prepared with the Oryx8 high-throughput crystallization robot (Douglas Instruments). UbC4 crystals were flash-frozen in liquid nitrogen and mounted onto cryoloops. Diffraction experiments were carried out at the Elettra-XRD2 beamline. The structure of UbC4 was solved by molecular replacement using the 1YF9 pdb entry (UbC4, space group: *P*4_3_2_1_2,^29^) as a model in MolRep 11.9.02 from the CCP4 9.0.010 program suite. Iteration cycles of refinement and model building were carried out using Refmac5 5.8.0430 and Coot 0.9.8.96. Figures were prepared with ICSF Chimera 1.18 and ChimeraX-1.9. Data collection and refinement statistics are reported in Table S1. Root-mean-square deviations (rmsd) between structures have been determined using the SUPERPOSE program in the CCP4 suite. The analysis of the protein cavities was performed using CastPFold (https://cfold.bme.uic.edu/castpfold/, Table S2).

### Structure-Based Fragment Screening

Crystallization plates were set up in our home laboratory using the Oryx4 robot from Douglas Instruments. UbC4 crystallized at 6.5 mg/mL in 3.1 M NaCl, 0.1 M MES/imidazole pH 6.5, 20% glycerol. At the XCHEM facility of Diamond Light Source, an ECHO acoustic liquid dispenser was first used to screen the maximum dimethyl sulfoxide (DMSO) concentration suitable for soaking. Then the ECHO instrument dispensed 733 fragments from the commercial DSi-Poised library (Enamine, provided by the XCHEM facility) onto the crystallization drops, resulting in a maximum concentration of 75 mM fragment and 15% DMSO.^30^ Soaked crystals were then incubated for 1 h at 20 °C. Crystals were rapidly mounted on cryoloops thanks to the OLT shifter^31^ and directly flash-frozen in liquid nitrogen prior to the diffraction experiments, which were carried out at the I04-1 beamline. Data processing was carried out using the XCHEM Explorer (XCE) pipeline.^32,33^ Hits were identified using Pan-Data set Density Analysis (PanDDA^34^), the refinement was performed by Buster,^35^ implemented on XCE. Table S3 reports the data collection and refinement statistics for the fragment screening experiment.

### Molecular Docking of Elongated Fragments

Crystallographic structures of UbC4 bound to fragments were used to predict promising elongated fragments using DeepFrag.^36^ Starting points for growing were set for the atoms of each fragment that lie the deepest in the cavities of interest (cavities 3 and 15). Candidates with a DeepFrag score higher than 0.6 were selected and used for molecular docking. AutoDock Vina 1.1.2 ^37,38^ implemented in UCSF Chimera 1.18 ^39^ was used to perform molecular docking on two distinct sites: cavity 15 for elongated fragments based on fragment 274 with coordinates *x* = −2, *y* = −24, and *z* = −41, size *x* = 12, size *y* = 12 and size *z* = 12 and cavity 3 for elongated fragments based on fragments 331, 360, 466, 637, and 733 with coordinates *x* = 10, *y* = −22, and *z* = −54, size *x* = 12, size *y* = 12 and size *z* = 12. Fragments identified by crystallographic fragment screening were also docked to evaluate the gain in predicted binding affinity of elongated fragments.

## Results and Discussion

### UbC4 Construct

In this study, we aimed to characterize a first set of enzymes belonging to the ubiquitin–proteasome system of *Leishmania* parasites and investigate their druggability: we utilized a truncated construct of UbC4 from *L. major* for several reasons. First, the crystallographic structure of the truncated UbC4 from *L. major* was already deposited in the Protein Data Bank (PDB: 1YF9) by the Structural Genomics of Pathogenic Protozoa Consortium (SGPP) in 2004. These structural data demonstrated that this construct is amenable to X-ray crystallography, a crucial prerequisite for reproducible crystallization and therefore high-throughput fragment screening. Second, attempts to express and purify the full-length UbC4 were unsuccessful, as it formed insoluble inclusion bodies. AlphaFold2 predictions (AF-Q4Q5L3-F1-v4,^40^) suggest that the full-length protein contains a long, unfolded C-terminal sequence (Figure S2), notably corresponding to the portion truncated off our construct. Such disordered regions often impede soluble expression, purification, and crystallization. More broadly, the structural characterization of proteins displaying substantial conformational flexibility remains a major challenge in X-ray crystallography, as conformational heterogeneity often prevents the formation of well-ordered crystals. This limitation is particularly relevant for proteins whose three-dimensional architecture and molecular mechanism have yet to be experimentally established, thereby slowing their functional characterization and the development of structure-guided approaches.

Finally, it is worth mentioning that sequence alignments show 98.2% sequence identity between UbC4 from *L. major* and that of *L. infantum*, i.e., the causative agent of Kala-azar (the most severe form of leishmaniasis), with only four substitutions, only two of which involve residues of different chemical nature (Figure S3).

### UbC4 Forms Thioester Complexes with Ub *In vitro*

We assessed the *in vitro* formation of the UbC4-Ub thioester bond at 37 °C in the presence of the putative E1 (UbA1a), Ub, and ATP over 4 h (Figure 1). Nonreducing SDS-PAGE (Figure 1A) revealed several additional bands of higher molecular weight compared to the UbA1a, UbC4, and Ub bands alone, which intensity vary over 4 h. Western blot analysis, exploiting the polyhistidine tag on Ub, clarified the nature of the observed bands (Figure 1B). The more intense band corresponds to the monoubiquitinated UbC4 complex (∼30 kDa). Additionally, a ∼22 kDa band, whose intensity increases overtime, corresponds to diubiquitin, Figure 1B, suggesting that UbC4 can facilitate uncontrolled Ub chain formation, a not so uncommon characteristic of E2s in the absence of an E3 ligase.^41,42^ Higher molecular weight bands likely represent polyubiquitinated UbC4 species, resulting from potential self-ubiquitination at surface-exposed lysine residues (*e.g*., K30, K45, K70, or K159, Figure S4). Of note, self-ubiquitination of E2s is a known mechanism involved in the regulation of E2s expression and activity.^43^ Among the polyubiquitinated UbC4 species, we can surmise that the bands account for the formation of UbC4-Ub_2_ (∼40 kDa), UbC4-Ub_3_ (∼50 kDa), and UbC4-Ub_4_ (∼60 kDa), while we can guess the presence of UbC4-Ub_5_ (∼70 kDa) and UbC4-Ub_6_ (∼80 kDa) based on the presence of some faint bands (Figure 1B,D).

**Figure 1:**
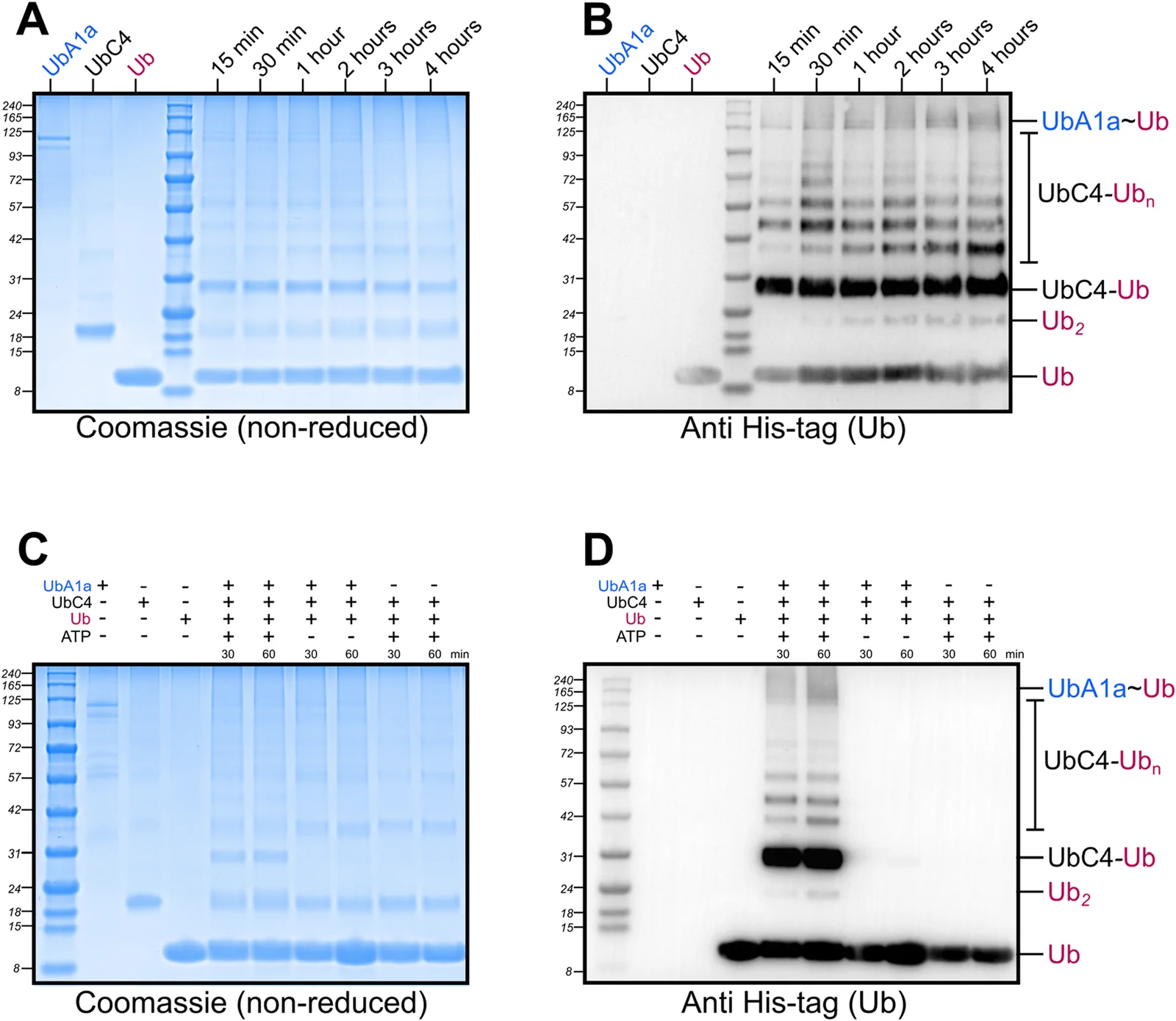
*In vitro* UbC4-Ub thioester bond formation in the presence of UbA1a and ATP. The UbC4-Ub thioester bond formation was carried out in the presence of UbA1a, UbC4, Ub and ATP at 37 °C and followed up to 4 h to track the enrichment of UbC4-Ub by SDS-PAGE (A) and Western blot (B), exploiting the purification His-tag attached to the recombinant Ub to identify Ub, diUb, mono- and polyubiquitinated species. The importance of UbA1a and the ATP-dependent nature of the thioester bond formation between UbC4 and Ub have been assessed by SDS-PAGE (C) and Western blot (D).

Interestingly, the band at ∼40 kDa, which corresponds to UbC4-Ub_2_, displays an increasing intensity over time (Figure 1B,D), suggesting the accumulation of this intermediate.

Additionally, the lack of bands corresponding to ubiquitinated species in the absence of UbA1a or ATP (Figure 1C,D) indicates that the process is ATP-dependent and requires E1 activation, supporting the functional role of the putative UbA1a, UbC4, and Ub in the *Leishmania* UPS.

### UbC4 Displays the Canonical E2 Ubiquitin-Conjugating Enzyme Fold

We solved the crystal structure of *L. major* UbC4 (LmUbC4) by molecular replacement at 2.16 Å resolution (Figure 2A). In this work, the reported residue numbering corresponds to that of the deposited structures of LmUbC4 (PDB: 1YF9 and 28HT). We observed two UbC4 monomers in the asymmetric unit. Although our truncated UbC4 construct contains 171 residues, we were able to reconstruct the polypeptidic chains only from Ser13 to Pro170 due to the poor electronic density describing the N- and C-terminal extremities, in agreement with the AlphaFold prediction that suggests that the N-terminal 10-residue segment is rather unstructured. Hereafter, we will describe the UbC4 monomer A, since both UbC4 in the asymmetric unit are nearly identical (rmsd = 0.352 Å, calculated with superpose, CCP4 software suite).

**Figure 2:**
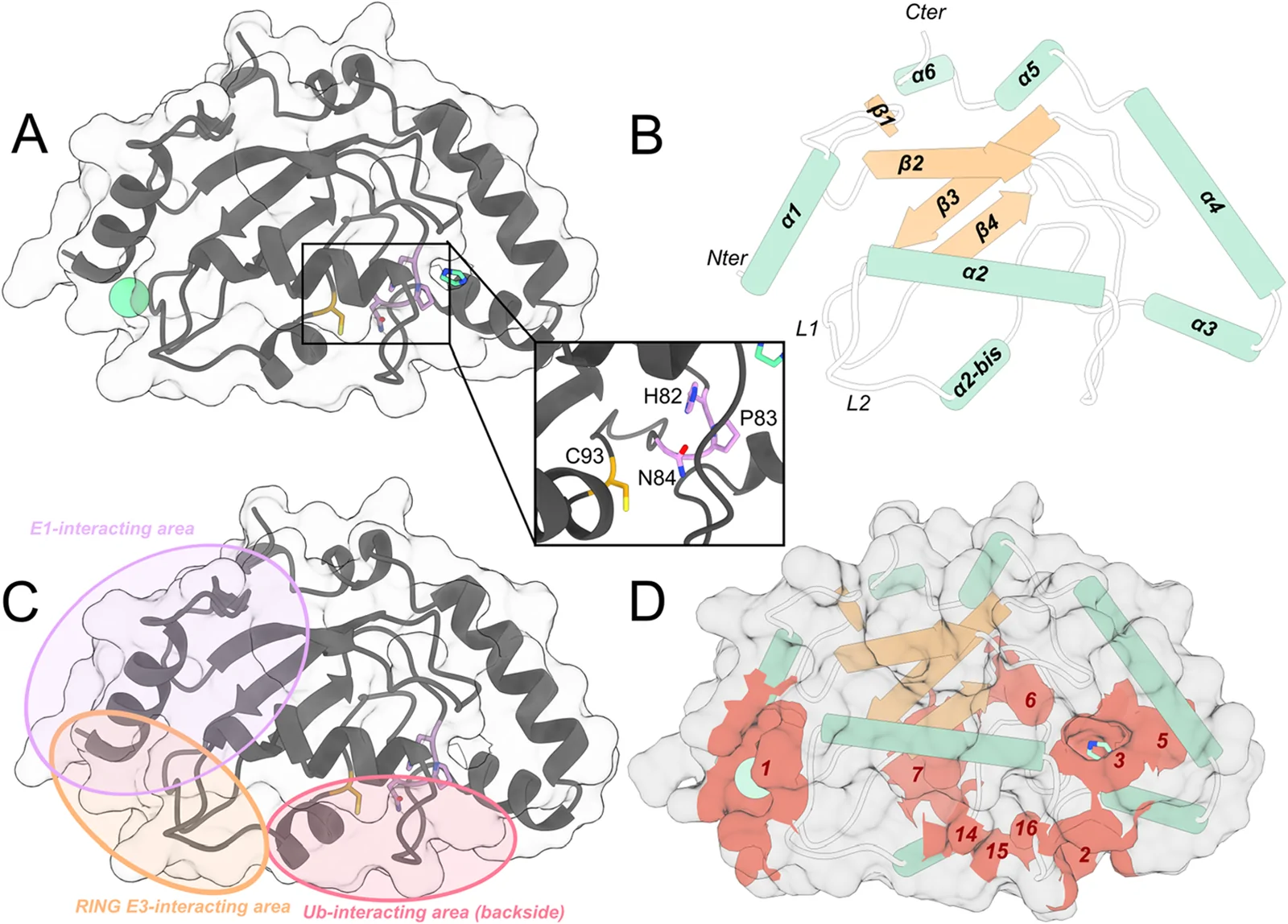
LmUbC4 displays the conserved Ubc fold. (A) Crystal structure of LmUbC4 with chloride ions and the imidazole belonging to the crystallization condition. Cys93 and the HPN triad located in the catalytic cleft are displayed in the close-up view (inset). (B) Analysis of the secondary structures of LmUbC4; α-helices are displayed as light green pipes while β-strands are shown as orange arrows. (C) Overall representation of LmUbC4 regions interacting with E1s, RING E3s, and Ub. These regions have been inferred for LmUbC4 based on previously deposited structures of the UbcH5b-Ub conjugate (3A33 pdb entry), the Ubc13 and TRAF6 (RING E3) complex (3HCT pdb entry), and Ubc12-NEDD8 E1 complex (2NVU pdb entry).^44,45^ (D) UbC4 cavities were identified using CastPFold (https://cfold.bme.uic.edu/castpfold/)^46^ (cavities 1, 2, 3, 5, 6 and 7 are the largest). For clarity, we also reported smaller pockets (14, 15 and 16) close to the catalytic cysteine. Secondary structures are represented as cartoons, except in panel (B), where α-helices and β-strands are shown as pipes and planks. Residues and imidazole molecules are displayed as sticks, and chloride atoms as large green spheres.

It is worth mentioning that the addition of glycerol in the crystallization conditions had an effect on crystal packing, as LmUbC4 in this study crystallized in the H32 space group, whereas the previously reported UbC4 from the Structural Genomics of Pathogenic Protozoa Consortium (SGPP,^29^ PDB: 1YF9) crystallized in the *P*4_3_2_1_2 space group, where the structure revealed three monomers in the asymmetric unit.

UbC4 is composed of six α-helices, a short 3_10_ helix (α2-bis), close to the putative catalytic cysteine, and four β-strands, forming a tertiary structure that resembles the canonical Ubc fold (Figure 2B), conserved among species. LmUbC4 aligns closely with HsUbE2H (PDB: 2Z5D, rmsd: 1.146 Å), its closest human homologue (Figure S5), and with HsUbE2K, a well-characterized E2 ubiquitin conjugating enzyme (Figure S6).^47^ The structure alignment with the above-mentioned human counterparts suggests that Cys93 is the catalytic cysteine.^48^ This cysteine, as expected for canonical E2s, is embedded in a groove between the β4-strand, the α2-helix and the short 3_10_ helix. Crucially, the HPN triad conserved among canonical E2s about 10 residues upstream of the catalytic cysteine is present in LmUbC4 (His82, Pro83 and Asn84, Figure 2A inset).^43^ Whereas the histidine is known to have a structural role in the active site, the asparagine is expected to mediate the catalytic formation of the isopeptide bond between the Ub and a substrate protein by stabilizing the oxyanion intermediate during the substrate lysine attack.^44,48–50^

To identify potential binding sites for inhibitors and E2-recruiting PROTACs with leishmanicidal properties, we first mapped potential surface cavities and pockets where organic compounds may accommodate using CastPFold (Figure 2D, Table S2).

The catalytic cleft (cavity 15) represents a natural target for the design of inhibitors, as well as any cavity whose occupation could allosterically affect catalytic activity.^51^ In contrast, noncatalytic cavities are more suitable targets for the development of PROTACs, as the E2 ubiquitination activity should not be hampered. In fact, the binding of a PROTAC ligand should not prevent the formation of the thioester bond between UbC4 and Ub or the access of Ub to Cys93. Additionally, the binding of a potential PROTAC should also not obstruct the interactions with either E1s or E3 ligases, as it would hamper Ub transfer to/from UbC4. Consistent with the conserved fold of canonical E2, our analysis indicates that E1/E3 interaction interfaces appear to be conserved: E1s typically engage the N-terminal region of E2s, whereas E3 ligases interact with the α1-helix and L1/L2 loops (Figure 2C).^44,45^

Notably, cavity 3 (the third largest pocket) shows great potential for PROTAC recruitment, for several reasons (Figure 2D): (i) selectivity: it is only partially conserved in human HsUbE2H but strictly conserved across the *Leishmania* genus (Figures S5 and S7), enabling the design of PROTACs specifically recruiting *Leishmania* UPS enzymes; and (ii) functional autonomy: it is far from key interactions sites with E1s, E3 ligases or Ub, suggesting that the binding of potential PROTACs would not interfere with the Ub transfer to the E2 and its ubiquitination activity. Indeed, cavity 3 is formed by the α3 and α4 helices, the α2–α3 loop, the C-terminal extremity of the β4–α2bis loop and the β2–β3 loop. Of note, in our crystal structure of apo-UbC4, cavity 3 hosts an imidazole molecule from the crystallization condition.

## Fragments Were Identified in Different Cavities of UbC4

We performed a crystallographic fragment screening campaign at the Diamond Light Source XCHEM facility, and we identified 10 fragments bound to different pockets of UbC4 (Figure 3 and Table 1). The fragments exhibited rather high real-space correlation coefficients (RSCC), except for fragment **116**. The RSCC measures how well the electron density of a modeled fragment matches the experimentally observed electron density, and although fragment **116** fell slightly below the typical 0.7 RSCC threshold, the PanDDA maps were sufficiently clear and unambiguous to support the inclusion of this fragment in the present analysis (Table S4). Interestingly, the most populated site was cavity 3, validating our initial computational mapping. In our UbC4 structure, this region hosts an imidazole molecule that arises from the crystallization conditions. Upon fragment binding, the resident imidazole is displaced and the pocket undergoes a localized induced-fit rearrangement at its entrance, with Arg140 and Asp144 moving aside to allow the full opening of the cavity to accommodate the ligands.

**Figure 3:**
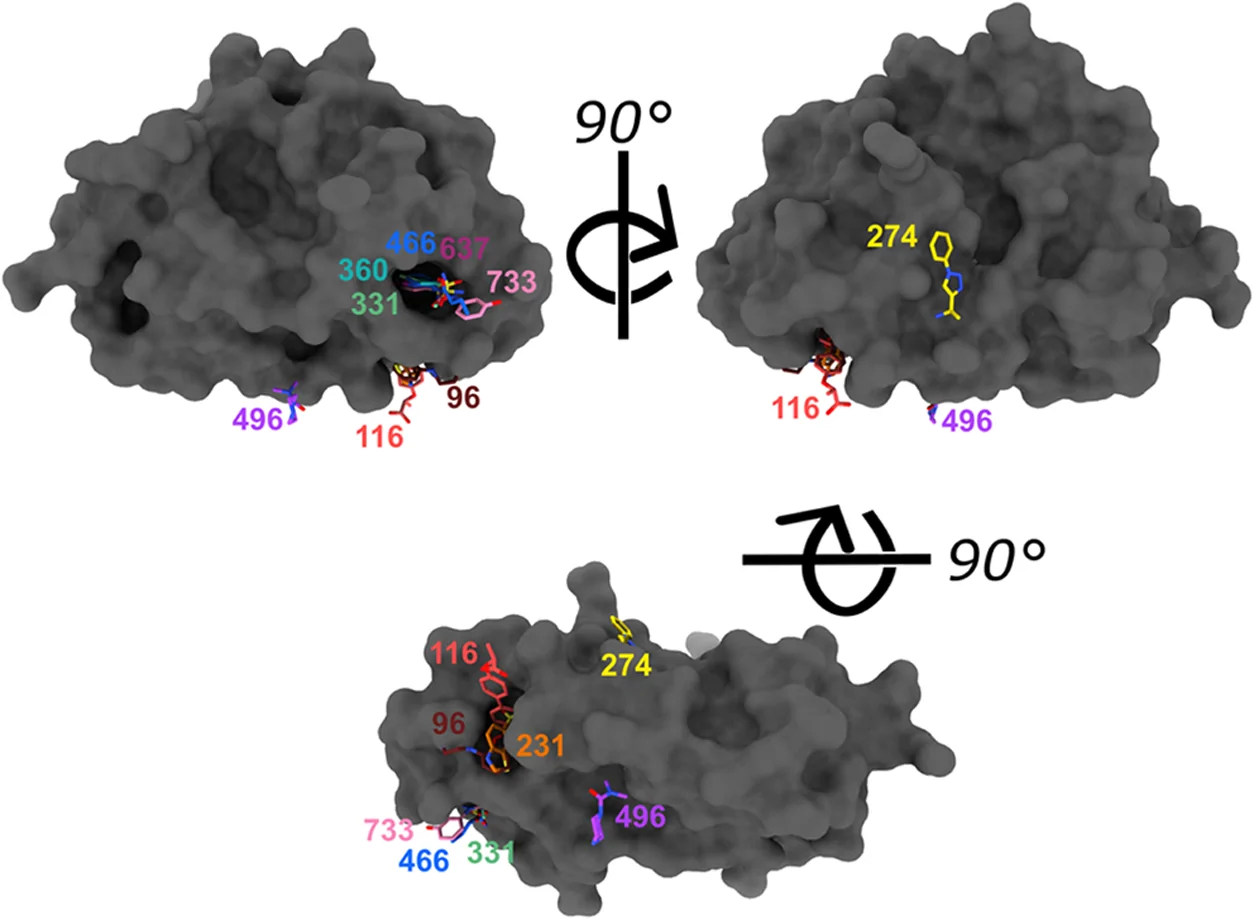
Crystallographic screening of the DSIpoised library on LmUbC4 crystals. Fragments are shown as colored sticks with their name in the corresponding color.

**Table 1:**
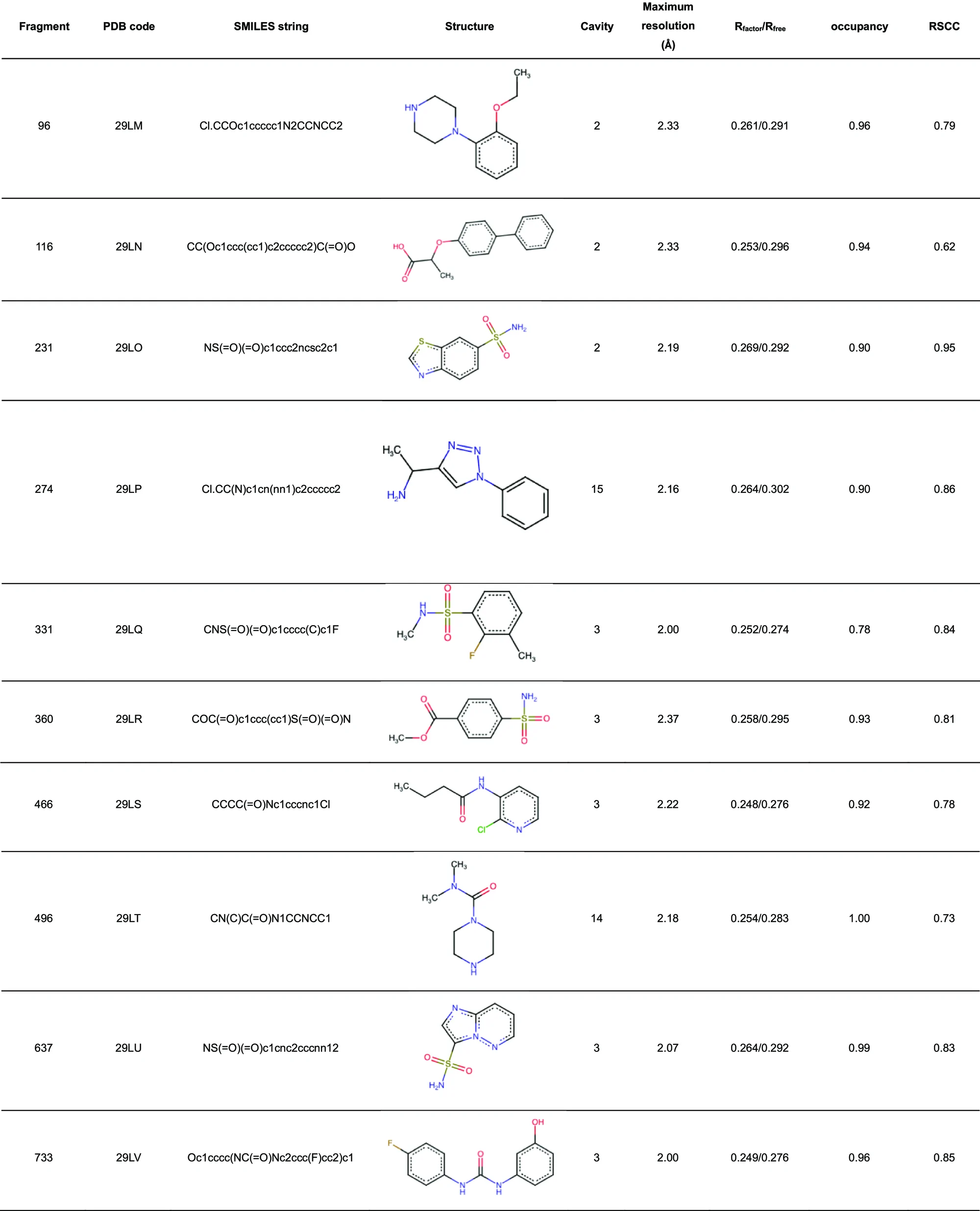
Fragment Details and Overall Data Quality

Notably, all five fragments identified in cavity 3, namely, **331**, **360**, **466**, **637**, and **733**, share a common binding fingerprint (Figure 4). In all cases, binding is driven by a planar aromatic or heteroaromatic moiety that inserts into a hydrophobic cleft defined by Tyr52, Leu136, Arg140, Phe143, and Leu147 (Table S5). This aromatic anchoring element is consistently coupled to a short polar linker, typically an amide or sulfonamide group, which establishes a conserved hydrogen bond with Asp144. The recurrence of this interaction across all fragments identifies Asp144 as a key hydrogen-bonding hotspot within the pocket. Within this shared binding mode, fragment **733** displays the most extensive polar interaction network, forming two H bonds with Asp144 through its diamide group, thereby reinforcing the central role of this residue in ligand recognition. An additional hydrogen bond with symmetry-related Asn26 is observed in the crystal lattice (Figure S8), although the conserved binding mode is primarily defined by direct interactions within the pocket.

**Figure 4:**
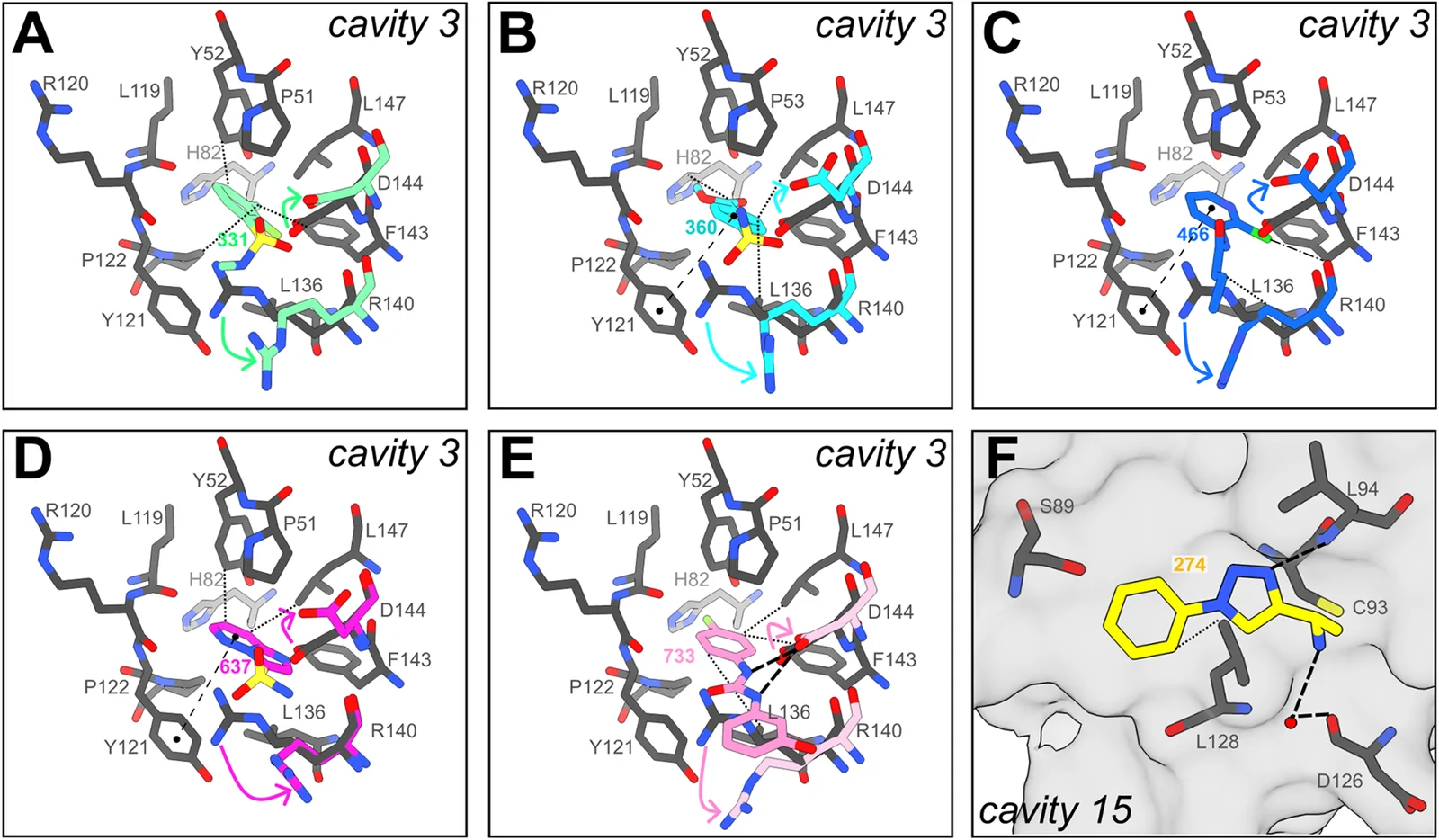
Fragments binding to cavities 3 and 15. The interactions between UbC4 and fragments 331 (A), 360 (B), 466 (C), 637 (D), 733 (E), and 274 (F) were identified using the Protein–Ligand Interaction Profiler (PLIP) Web server (https://plip-tool.biotec.tu-dresden.de/ ^52^) and they are displayed as dotted lines for hydrophobic interactions, as dashed lines with end points for π–π interactions, as strong dashed lines for H-bonds and electrostatic interactions, and as mixed dashed-dotted lines for halogen bonds. UbC4 residues are shown in dark gray except H82 belonging to the HPN motif colored in lighter gray. Residues and fragments are represented as sticks. The displacement of Arg140 and Asp144 in cavity 3 with respect to UbC4 in the absence of fragments is indicated by a colored arrow.

However, the depth reached by the aromatic moieties slightly differs among the fragments, depending on the presence of additional chemical substituents that affect steric hindrance, indicating that peripheral groups modulate the extent of pocket occupancy without altering the core binding mode. For example, the bulky methyl ester substituent on fragment **360** restricts the deeper penetration of the aromatic ring observed for the other fragments. Taken together, these data identify cavity 3 as a ligand-responsive noncatalytic pocket with a well-defined recognition pattern, providing a promising starting point for the design of compounds capable of recruiting this E2 from *Leishmania*. As expected for fragment hits, the identified ligands are likely endowed with low affinity; however, the structural data suggest promising opportunities for fragment growth, particularly by elaborating the moieties projecting into the deepest region of cavity 3, with the aim of improving both affinity and specificity for UbC4.

Interestingly, we identified fragment **274** in cavity 15, i.e., the shallow cavity containing the catalytic Cys93 (Figure 4F). Although cavity 15 lies near symmetry-related copies of UbC4, the closest group is the terminal guanidinium of symmetry-related Arg79, located more than 4 Å from the C8 atom of fragment **274** aromatic ring, therefore not participating to strong interactions and suggesting that crystal contacts do not significantly contribute to the stabilization of **274** in this pocket. Instead, fragment **274** is stabilized by a combination of an electrostatic interaction with Leu94, a water bridge to Asp126, and a hydrophobic interaction with Leu128 (Figure 4F). Its proximity to Cys93 makes fragment **274** an ideal starting point for the design of inhibitors to potentially hamper the UbC4-Ub thioester formation.

Some small-molecule inhibitors of human E2s were already reported in the scientific literature. For example, the 2α,6α-diacetoxy-4β-hydroxy-11(13)-pseudoguaien-12,8α-olide (DHPO) compound was predicted to form H-bonds with Leu86 and Arg90 of UbcH5c, in the close vicinity of the catalytic Cys85.^51^ A surprising example is the CC0651 inhibitor designed for the human Cdc34 ubiquitin conjugating enzyme. This inhibitor does not directly hamper the activity of the cysteine but triggers a large deleterious structural rearrangement upon binding of CC0651 through its accommodation into a cavity, 19 Å away from the catalytic cysteine and newly formed in the presence of CC0651, that probably alters the ubiquitin transfer reaction.^50^

The fragments **96**, **116**, and **231** were found in cavity 2, while fragment **496** was found in cavity 14. These fragments, although identified in interesting nonenzymatic cavities, are to be considered with caution, since their presence inside these grooves seems to be due partly to interactions with crystal symmetric copies of UbC4, suggesting that in solution such fragments may not display the same binding propensity as in the crystalline state, aided by the crystal symmetry contacts (Figure S9).

### Structure-Guided Fragment Elongation

To transition from low-affinity fragments to higher-affinity leads, we employed DeepFrag, a deep-learning tool designed to suggest chemically feasible modifications for fragment growth.^36^ We focused on fragments **331**, **360**, **466**, **637**, and **733** in cavity 3 that represent suitable starting points for the design of E2-recruiting PROTACs, and fragment **274**, binding to cavity 15, which could be grown into inhibitors by masking the catalytic cysteine. In line with the structural analysis of the binding pockets, we focus on the fragment atoms located deeper in the cavities as potential growing points. The resulting compounds were then docked into the cavities of interest of UbC4 using AutoDock vina 1.1.2.^37,38^ For comparison, we also docked the starting fragments identified during the crystallographic fragment screening campaign. The modifications proposed by DeepFrag and the corresponding docking scores are reported in Figure 5. It is worth mentioning that, at the exception of **360**, **637**, and **733**, fragments are endowed with rather low scores (>6 kcal/mol) and limited improvement upon elongation, while maintaining physicochemical properties compatible with further optimization (Table S6).

**Figure 5:**
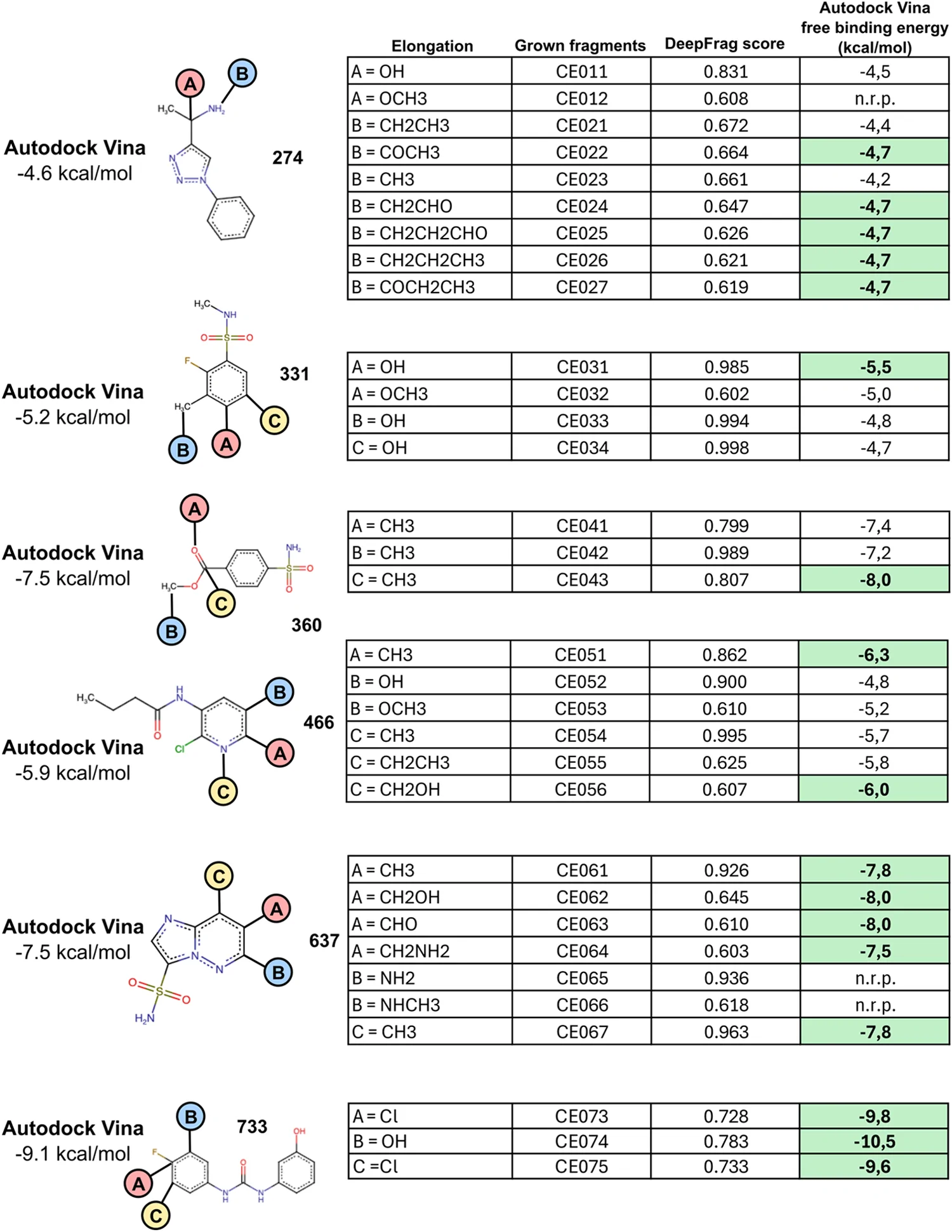
Molecular docking of fragments predicted by DeepFrag. The initial fragments identified by crystallographic fragment screening are shown on the left. The letters A, B, C, and D indicate the growing points for the functional groups predicted by DeepFrag, and the DeepFrag confidence score is reported in the first column of the table. The last column reports the results of the molecular docking campaign carried out with AutoDock vina 1.1.2 implemented in UCSF Chimera 1.18. Improvement of the docking score of elongated fragments compared to the original ones is highlighted in green. Non-reliable poses (nrp) indicate docking calculations providing only incoherent poses compared to those of the crystallography-identified fragments. SMILES strings and a preliminary in silico absorption, distribution, metabolism, excretion, and toxicity (ADMET) analysis for each DeepFrag prediction are reported in Table S6.

Fragment **733** displays the highest binding free energy, and its derivative **CE074**, featuring a hydroxyl group on one of the carbons of the fluoro-phenyl ring, results in a further improvement of the binding energy by ∼1.5 kcal/mol. This modification allows the fragment to extend slightly deeper into cavity 3, forming new electrostatic interactions with His82 and Arg120 (Figure 6A).

**Figure 6:**
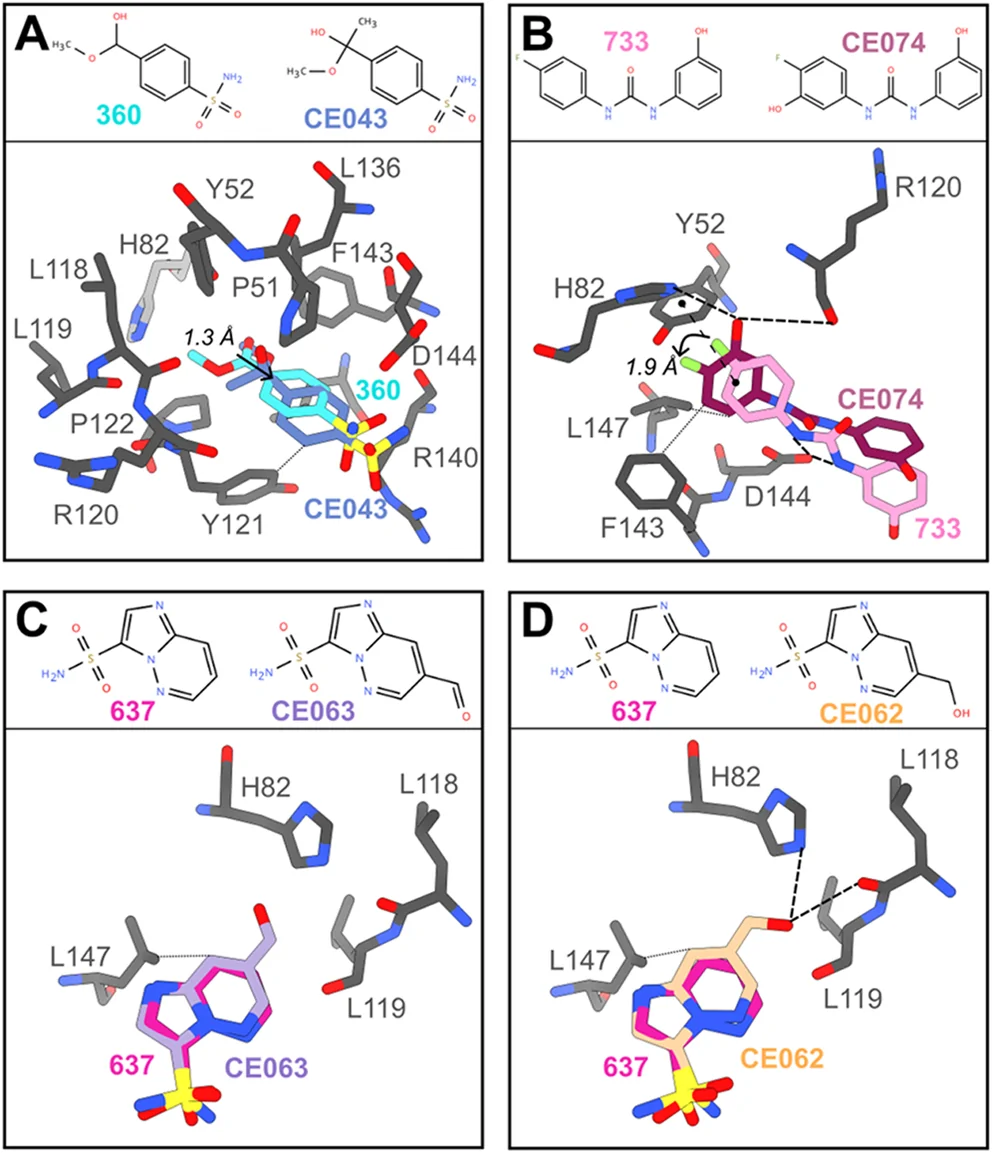
Molecular docking of **CE043**, **CE062**, **CE063**, and **CE074** into cavity 3. The interactions between UbC4 and elongated fragments were identified using the Protein–Ligand Interaction Profiler (PLIP) Web server (https://plip-tool.biotec.tu-dresden.de/ ^52^), and they are displayed as dotted lines for hydrophobic interactions, as dashed lines with end points for π-stacking, and as strong dashed lines for H-bonds and electrostatic interactions. The displacement of compounds is indicated by a black arrow. Residues and compounds are displayed as sticks. UbC4 residues are shown in dark gray.

Similarly, fragments **360** and **637** show slight improvements in the docking score (0.5 kcal/mol) upon elongation. **CE062** and **CE063**, derived from fragment **637** by the addition of an aldehyde and a hydroxymethyl group, respectively (Figure 5), retain binding poses of **637** observed by X-ray crystallography, while showing modest improvement in docking scores (Figure 6C,D).

Interestingly, the addition of a methyl group on the carbonyl carbon of **360**, yielding compound **CE043**, increases steric bulk and appears to hinder the deep accommodation of the phenyl ring into cavity 3 (Figure 6B).

## Conclusion

Leishmaniases remain neglected diseases that affect around 1 million people each year. Most available treatments are either highly toxic or prone to resistance, highlighting the urgent need for new therapeutic options that overcome these limitations. In this context, PROTACs could represent a viable strategy, as they have the potential to reduce both toxicity and resistance issues. However, very little is known about the ubiquitin–proteasome system (UPS) in *Leishmania*. More particularly, only two E3 ligases bearing a CULT domain have been identified, and none with a von Hippel–Lindau (VHL) domain,^13^ which is the classical E3 ligase recruited by clinically advanced PROTACs.

Our work provides an important step forward in understanding the UPS of *Leishmania* that we identify as a viable target, specifically proposing the E2 ubiquitin-conjugating enzyme, LmUbC4, as a novel PROTAC recruitment module. This contrasts with traditional PROTACs that typically recruit E3 ligases such as VHL or CRBN, which appear to be absent or uncharacterized in *Leishmania*.

By characterizing the structural landscape of LmUbC4 and identifying druggable pockets, we provided a roadmap for the development of both specific E2 inhibitors and E2-recruiting PROTACs. The identification of cavity 3 as a selective, ligand-responsive site offers a unique opportunity to engage the *Leishmania* UPS with high specificity.

In more detail, we demonstrated the role of LmUbC4 as an E2 ubiquitin-conjugating enzyme following the formation of thioester bonds with ubiquitin (Ub), and we observed that it can be mono- and self-polyubiquitinated in the presence of UbA1a (E1) and Ub from *L. infantum*. The conserved fold of UbC4, as evidenced by X-ray crystallography, support its role and implication in the *Leishmania* ubiquitin proteasome system (UPS). UbC4 displays several druggable cavities, which we partially mapped with a crystallographic fragment screening campaign that yielded the identification of 10 fragments binding to different cavities of the E2. Interestingly, 5 of them were observed in a partially conserved noncatalytic cavity (cavity 3). Such fragments could be optimized into ligands to design PROTACs against *Leishmania* essential enzymes like trypanothione reductase, paving the way toward the design of E2-recruiting PROTACs.

Additionally, we identified a fragment binding in a cavity close to the catalytic Cys93 that is competent for the formation of the covalent bond with Ub. Optimization efforts on this fragment could lead to the design of a potent E2 inhibitor by masking the cysteine and hampering the formation of the thioester bond with Ub.

We also provide the first attempts at fragment optimization using DeepFrag, a computational tool to provide inputs for the elongation of small molecules compatible with the protein of interest, i.e. UbC4 in the present work. Elongated fragments were docked, and improved binding energies were predicted compared to those of initial fragments. Overall, these results highlight chemically plausible growth vectors consistent with the structural features of cavity 3, supporting the feasibility of structure-guided optimization, while underscoring the need for experimental validation.

## Supplementary Material



## Notes

Scheme of the ubiquitination pathway (Figure S1); the AlphaFold prediction of full length UbC4 structure (Figure S2); the sequence alignments between Ubc4 from *L. major* and homologues from the *Leishmania* genus (Figure S3); the crystallographic statistics for the structure of UbC4 (Table S1); the mapping of UbC4-exposed lysines (Figure S4); the sequence alignment between Ubc4 from *L. major* and the closest homologue from human (Figure S5); the superimposition of LmUbC4 and HsUbE2K (5DFL(2)) structures (Figure S6); information on the mapped cavities of UbC4 (Table S2); the mapping of the imidazole cavity in UbC4 (Figure S7); the crystallographic statistics regarding the fragment screening experiment (Table S3); the PanDDA map analysis of the fragment screening experiment (Table S4); the physicochemical classification of amino acid residues interacting with each selected fragment (Table S5); the analysis of the involvement of the symmetric Asn26 in the stabilization of fragments from cavity 3 (Figure S8); a figure showing the analysis of stabilizing interactions of fragments 96, 116, 231, and 496 (Figure S9); the *in silico* ADMET analysis of predicted elongated fragments (Table S6) (PDF)

## Abbreviations

PROTACs: proteolysis targeting chimeras
Ub: ubiquitin
UbA1a: ubiquitin activating enzyme 1a (E1)
UbC4: ubiquitin conjugating enzyme 4 (E2)
UPS: ubiquitin proteasome system
