# Supplementary material for: UbC4 from Leishmania is a Druggable E2 Ubiquitin Conjugating Enzyme: Structural Basis and Fragment Hits for Future E2-Recruiting PROTAC Development
Source: ACS Omega. 2026 Sep 8;11(37):55759–71. doi: 10.1021/acsomega.6c05158 (PMC13613956; doi:10.1021/acsomega.6c05158)
Supplement: Supplementary file 1 [file ao6c05158_si_001.pdf]

Supporting Information

UbC4 from Leishmania is a Druggable E2 ubiquitin conjugating enzyme: Structural Basis and Fragment Hits for Future E2-Recruiting PROTAC Development

Cécile Exertier[1], Lorenzo Antonelli[1], Anastasia Liuzzi[1], Marcus Ruffa[1,2], Vittorio Brufani[1,2], Gianni Colotti[1], Annarita Fiorillo[1,2] & Andrea Ilari[1]*.*

[1]Institute of Molecular Biology and Pathology, Italian National Research Council (IBPM-CNR), c/o Department Biochemical Sciences, Sapienza University of Rome, Ed. CU027, P.le A.Moro 5, 00185 Rome, Italy;

[2]Department Biochemical Sciences, Sapienza University of Rome, Ed. CU027, P.le A.Moro 5, 00185 Rome, Italy

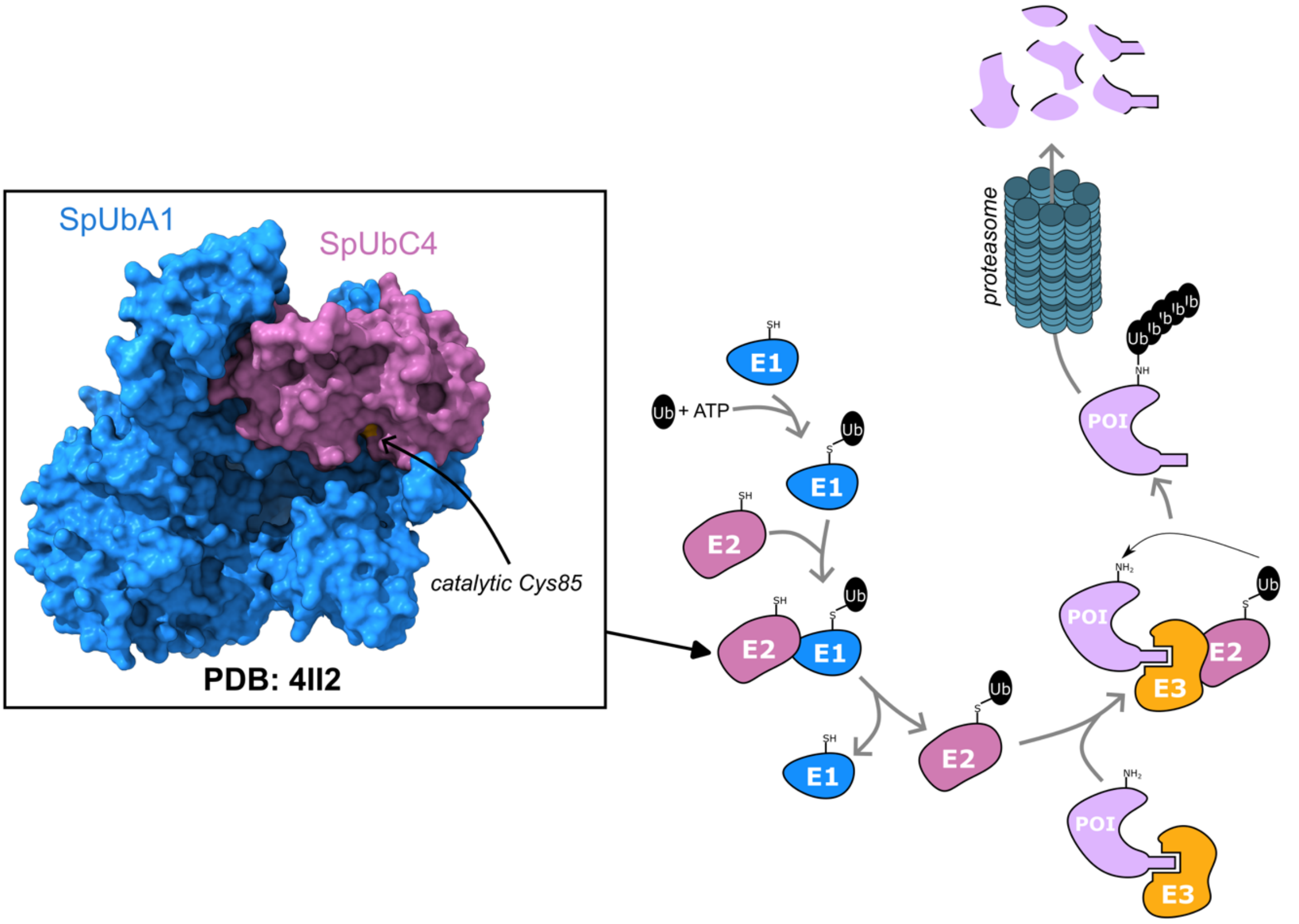

Figure S1. Ubiquitination casacade. Briefly, The E1 (UbA1) activates the ubiquitin (Ub) through an ATP-dependent adenylation of its C-terminal carboxyl group, enabling the formation of a thioester bond between the E1 and Ub. Ub is then transferred to the catalytic cysteine of an E2 through a trans-thioesterification thanks to the formation of a transient E1-E2 complex. An example of E1-E2 complex from *Schizosaccharomyces pombe* is shown in the insert on the left, which structure has been solved by X-ray crystallography (PDB: 4II2 (1)). From there, RING E3 ligases promote the direct transfer of Ub from an E2-Ub to a substrate lysine.

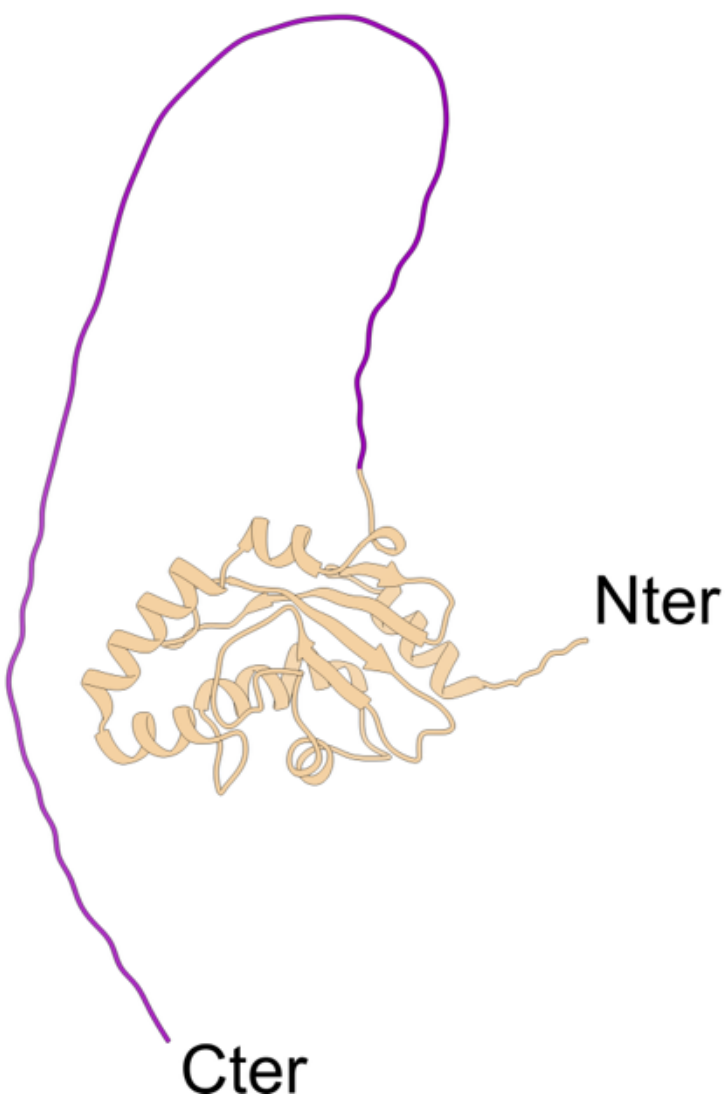


```
        10         20         30         40         50
MSGAG NLRSN RRREM DYMRL CNSTR KVYPS DTVAE FWVEF KGPEG TPYED

        60         70         80         90        100
GTWML HVQLP SDYPF KSPSI GFCNR ILHPN VDERS GSVCL DVINQ TWTPM

       110        120        130        140        150
YQLEN IFDVF LPQLL RYPNP SDPLN VQAAH LLHAD RVGFD ALLRE HVSTH

       160        170        180        190        200
ATPQK ALESI PEAYR PHDNT HEEEP DEANA KDTAS DGPVN AGHSK GLTDS

       210        220        230
SVTPT EDHRV MHDDI AIDGD EDVEA EYEPE EIDL
```

Figure S2. AlphaFold prediction of full length UbC4 from *Leishmania major*. The structure on the left has been predicted by AlphaFold for the full length UbC4 (AF-Q4Q5L3-F1-v4 (40)). The truncated construct of UbC4 expressed, purified and crystallized in this study is show in light orange, while the truncated portion is shown in dark purple both on the AlphaFold prediction and on the sequence on the right.

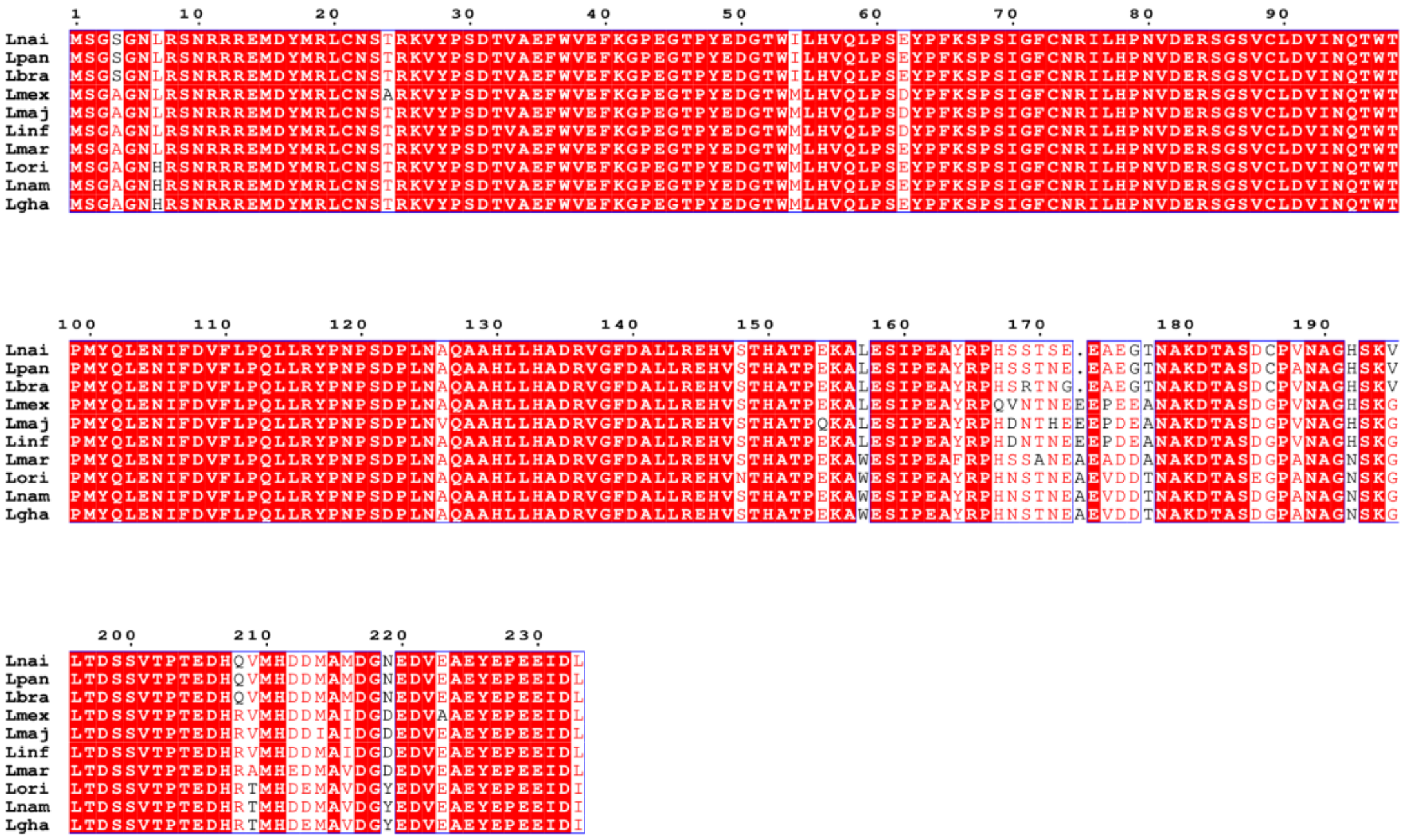

Figure S3. UbC4 Sequence alignment: *Leishmania* strains. These sequences were identified using BLAST (https://blast.ncbi.nlm.nih.gov/Blast.cgi)

Table S1. Crystallographic data collection and refinement statistics for the crystal structure of UbC4. The statistics for the last resolution shell are reported into parentheses.

	**UbC4**
PDB code	28HT
**Data Collection**	
Resolution range	83.228 – 2.157 (2.194 – 2.157)
Beamline	ELETTRA-XRD2
Wavelength	0.99990
Oscillation range	0.5
Number of images	720
Space group	H32
Cell dimensions	115.00/115.00/151.54
Cell dimensions	90.0/90.0/120.0
Observed reflections	380917 (20603)
Unique reflections	20142 (1051)
Multiplicity	18.9 (19.6)
Completeness	95.9 (100.0)
Mean I/sigma	20.1 (0.9)
CC1/2	0.998 (0.357)
Rmerge	0.089 (4352)
**Refinement**	
Resolution range	83.228 – 2.157
Rf/Rfree	0.19/0.25
Number of reflections	19007
Atoms for protein (MONA/MONB)	1306/1308
Atoms for solvent/waters	30/54
Bfactor for protein (MONA/MONB)	54.2/84.2
Bfactor for solvent/water	69.3/61.1
Rms bonds	0.0055

Rms angles	1.6056
Ramachandran favored (%)	95.2
Ramachandran allowed (%)	4.8
Ramachandran outliers (%)	0.0

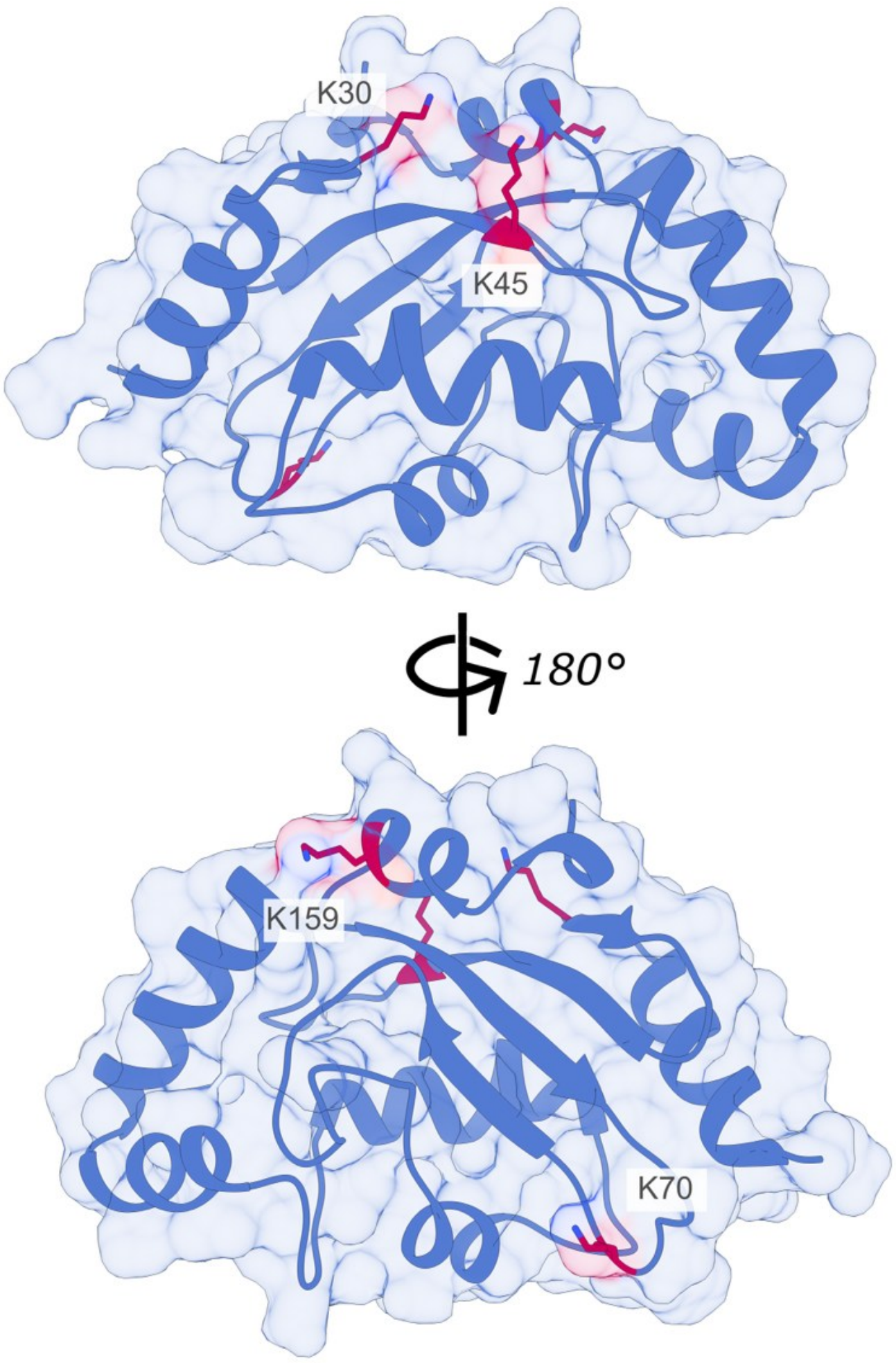

Figure S4. Exposed lysines of UbC4. UbC4 contains 4 lysines, all of them exposed to the solvent. The ubiquitination assay suggests UbC4 self-ubiquitination, that may happen on these lysine residues.

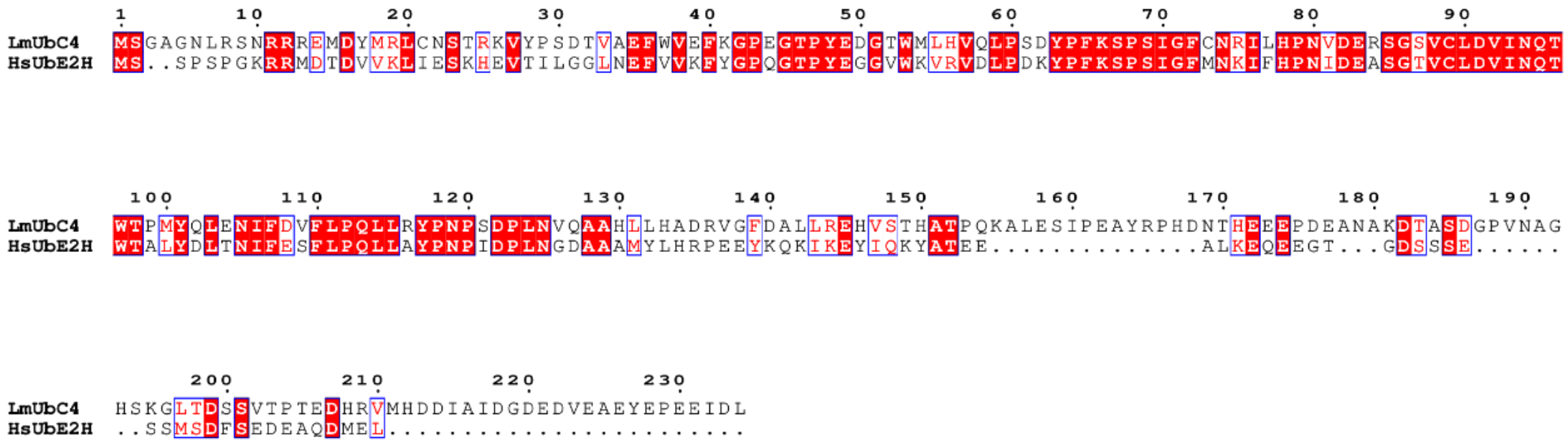

Figure S5. UbC4 Sequence alignment: *Leishmania major* vs. *Homo sapiens*. The closest human E2 homolog is HsUbE2H (Uniprot P62256). The homolog was identified using BLAST (https://blast.ncbi.nlm.nih.gov/Blast.cgi) and displays a 52.7% sequence identity with UbC4 from *Leishmania major*.

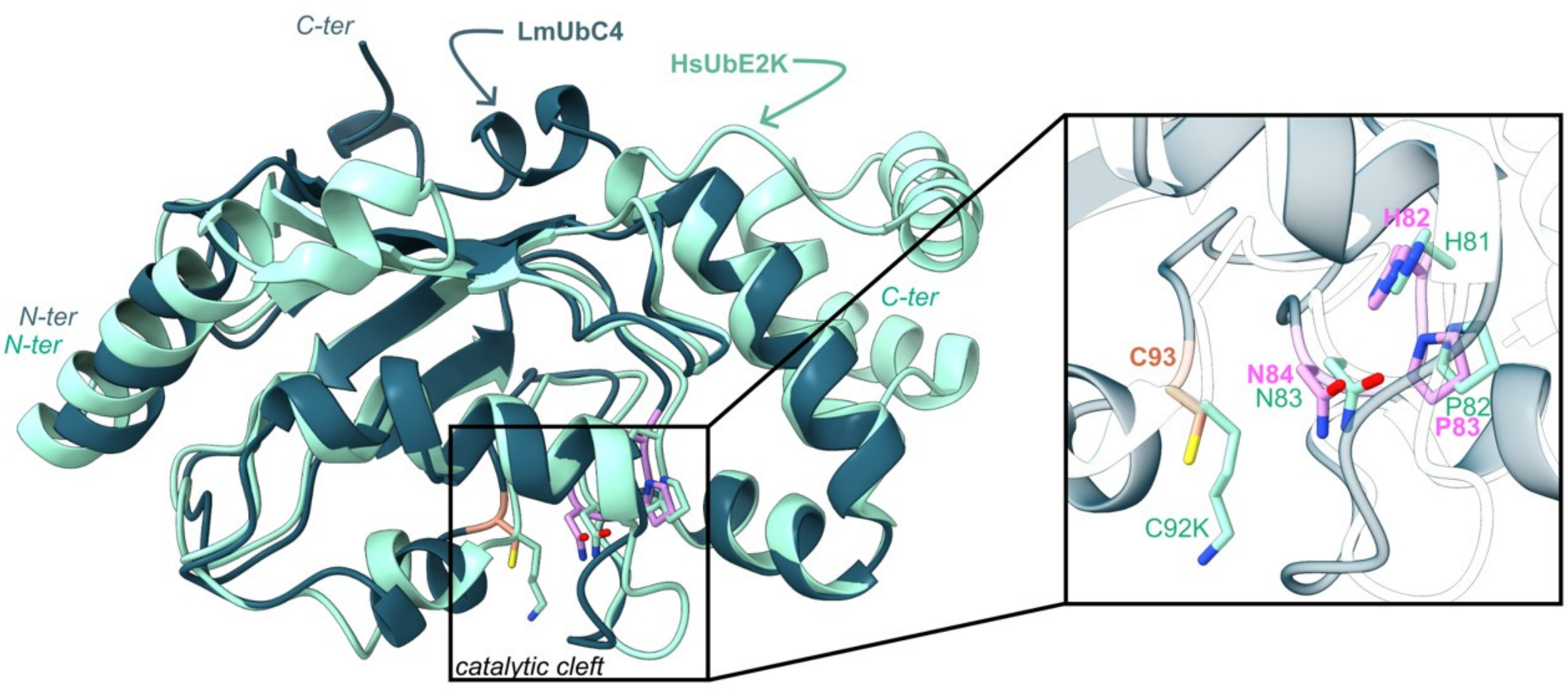

Figure S6. Superimposition of LmUbC4 and HsUbE2K (5DFL(2)) structures. The comparison shows the conservation of the Ubc fold, and the position of the catalytic cysteine and HPN triad in UbC4. It is worth mentioning that the HsUbE2K structure deposited on the PDB under the accession number 5DFL comes from a C92K mutated construct.

Table S2. Main cavities of UbC4 identified by CastPFold.

Cavities number	MS volume (Å^3)	Pocket MS area (Å^2)	Ligand
1	312.2	260.6	Chloride ion
3	178.5	172.0	Imidazole
2	136.8	111.6	-
6	68.1	76.1	-
5	60.5	70.8	-
7	59.1	79.5	-
16	19.5	35.8	-
15	10.6	18.5	(close to Cys93)
14	9.1	15.8	-

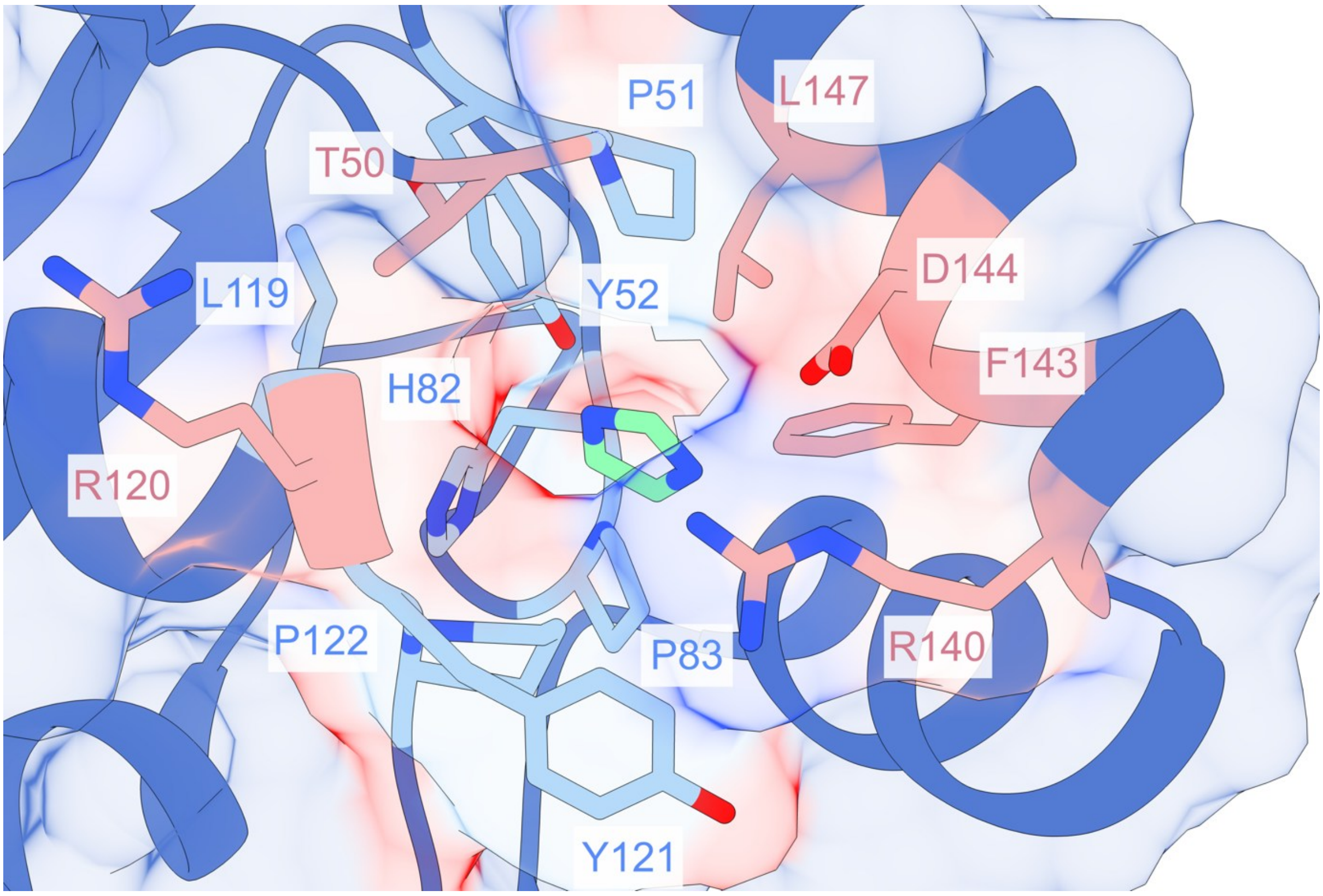

Figure S7. The "imidazole" cavity is partially conserved in HsUbE2H the closest human homolog of UbC4. Conserved residues are shown in blue, whereas non-conserved residues are shown in light red. Secondary structures are shown in cartoon representation. Residues and the imidazole are displayed as sticks. Notably, the Arg140–Asp144 pair, located at the entrance of the cavity, is not conserved in the human homolog. In UbC4, these residues form a salt bridge in the absence of ligands, which is not observed in the fragment-bound structures.

Table S3. Crystallographic data collection and refinement statistics for the fragment screening. The statistics for the last resolution shell are reported into parentheses.

Fragment	96	116	231	274	331
PDB	29LM	29LN	29LO	29LP	29LQ
Compound code	Z104474512	Z111782404	Z1269184613	Z1428159350	Z1639162606
SMILES string	Cl.CCOc1ccccc1N2CCNCC2	CC(Oc1ccc(cc1)c2ccccc2)C(=O)O	NS(=O)(=O)c1ccc2ncsc2c1	Cl.CC(N)c1cn(nn1)c2ccccc2	CNS(=O)(=O)c1cccc(C)c1F
**Data collection**					
Resolution range	84.37 – 2.33 (2.37 – 2.33)	84.28 – 2.33 (2.37 – 2.33)	84.41 – 2.19 (2.23 – 2.19)	84.32 – 2.16 (2.20 – 2.16)	84.27 – 2.00 (2.04 – 2.00)
Beamline	Diamond-I04-1	Diamond-I04-1	Diamond-I04-1	Diamond-I04-1	Diamond-I04-1
Wavelength	0.921344	0.921344	0.921344	0.921344	0.921344
Space group	H32	H32	H32	H32	H32
Cell dimensions	115.87/115.87/155.53	116.11/116.11/154.20	115.69/115.69/156.32	115.93/115.93/154.96	115.83/115.83/154.92
Cell dimensions	90°/90°/120°	90°/90°/120°	90°/90°/120°	90°/90°/120°	90°/90°/120°
Unique reflection	17405 (1698)	17350 (1685)	20955 (1799)	21692 (1871)	27205 (1999)
Multiplicity	20.6 (20.2)	20.6 (20.1)	20.6 (21.0)	20.5 (20.9)	20.4 (17.7)
Completeness (%)	100.0 (99.1)	100.0 (99.4)	100.0 (100.0)	100.0 (99.8)	100.0 (100.0)
Mean I/sigma	14.7 (0.2)	14.3 (0.3)	14.5 (0.2)	14.3 (0.2)	21.3 (0.3)
CC1/2	1.0 (0.27)	0.99 (0.42)	1.0 (0.25)	1.0 (0.26)	1.0 (0.39)
Rmerge	109 (6831)	111 (6591)	115 (6453)	98 (6009)	67 (4729)
**Refinement**					
Rf/Rfree	0.261/0.291	0.253/0.296	0.269/0.292	0.264/0.302	0.252/0.274
Bfactor for protein (MONA/B)	98.5/149.9	85.7/136.1	79.2/112.1	77.8/126.8	71.0/115.6
Bfactor for ligand	105.3	109.2	53.5	106.4	91.5
Ramachandran outliers (%)	0	0.32	0	0	0

Fragment	360	466	496	637	733
PDB	29LR	29LS	29LT	29LU	29LV
Compound code	Z18197050	Z240654968	Z270760338	Z3765495818	Z55290386
SMILES string	COC(=O)c1ccc(cc1)S (=O)(=O)N	CCCC(=O)Nc1cccnc1Cl	CN(C)C(=O)N1CCNCC1	NS(=O)(=O)c1cnc2 cccnn12	Oc1cccc(NC(=O)Nc2ccc(F) cc2)c1
**Data collection**					
Resolution range	61.40 – 2.37 (2.41 – 2.37)	61.19 – 2.22 (2.26 – 2.22)	84.18 – 2.18 (2.22 – 2.18)	83.71 – 2.07 (2.11 – 2.07)	83.99 – 2.00 (2.04 – 2.00)
Beamline	Diamond-I04-1	Diamond-I04-1	Diamond-I04-1	Diamond-I04-1	Diamond-I04-1
Wavelength	0.921344	0.921344	0.921344	0.921344	0.921344
Space group	H32	H32	H32	H32	H32
Cell dimensions	115.84/115.84/155.20	115.81/115.81/154.38	115.86/115.86/154.28	115.49/115.49/152.53	115.81/115.81/153.21
Cell dimensions	90°/90°/120°	90°/90°/120°	90°/90°/120°	90°/90°/120°	90°/90°/120°
Unique reflection	16491 (1694)	19922 (1776)	21004 (1786)	24048 (1853)	26837 (1934)
Multiplicity	20.7 (21.8)	20.6 (20.7)	20.5 (20.9)	20.6 (20.0)	20.4 (17.6)
Completeness (%)	99.9 (98.5)	100.0 (99.3)	100.0 (100.0)	100.0 (99.8)	100.0 (99.7)
Mean I/sigma	16.0 (0.3)	20.0 (0.4)	11.1 (0.3)	17.3 (0.2)	18.4 (0.3)
CC1/2	1.0 (0.49)	1.0 (0.39)	1.0 (0.34)	1.0 (0.37)	1.0 (0.38)
Rmerge	127 (6802)	82 (4626)	121 (4985)	83 (5461)	75 (4948)
**Refinement**					
Rf/Rfree	0.258/0.295	0.248/0.276	0.254/0.283	0.264/0.292	0.249/0.276
Bfactor for protein	91.0/142.2	79.2/123.9	73.1/115.5	75.5/118.1	71.1/110.0
Bfactor for ligand	98.1	102.1	113.3	83.6	98.3
Ramachandran outliers (%)	0	0	0	0	0

Table S4. Density maps obtained from the PanDDA analysis.

Fragment	PDB ID	Fragment - structure and nomenclature	PanDDA maps: Event maps (1 σ) z-maps (2.5 σ)	Density maps: 2Fo-Fc maps (1 σ) and Fo-Fc maps (3 σ)
96		**Structure:** 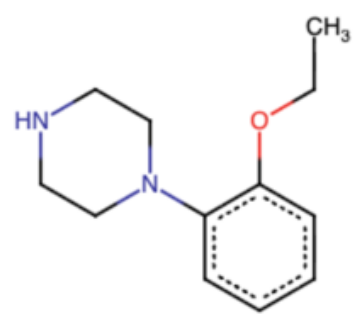 **SMILES string:** Cl.CCOc1ccccc1N2CCNCC2 **Name:** Z104474512	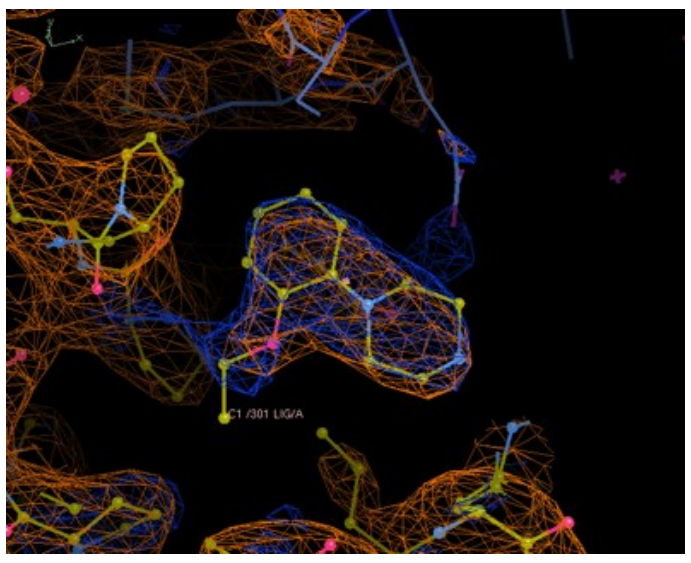	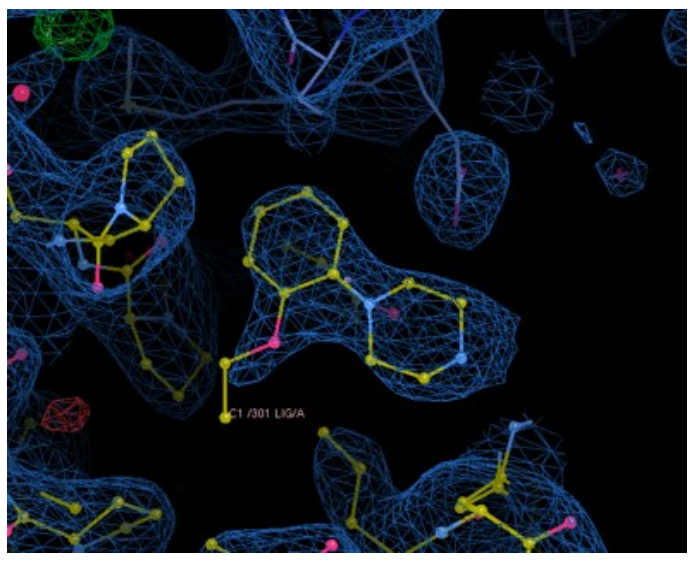
116		**Structure:** 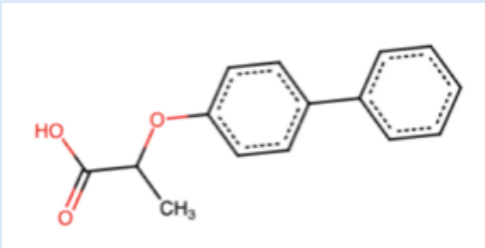 **SMILES string:** CC(Oc1ccc(cc1)c2ccccc2)C(=O)O **Name:** Z111782404	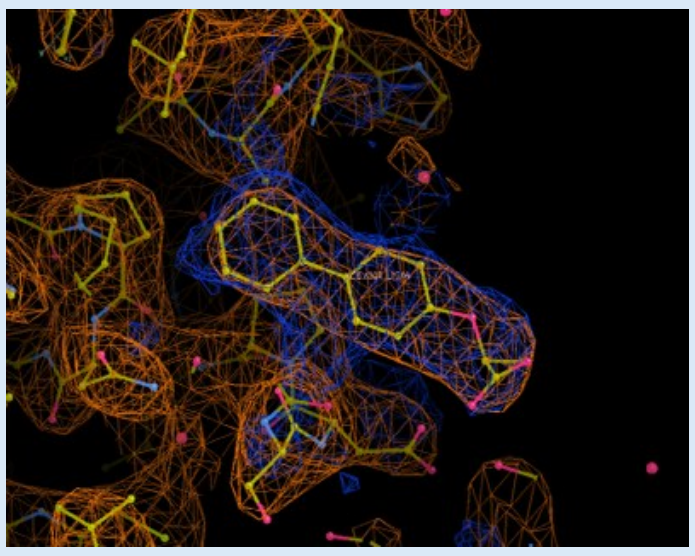	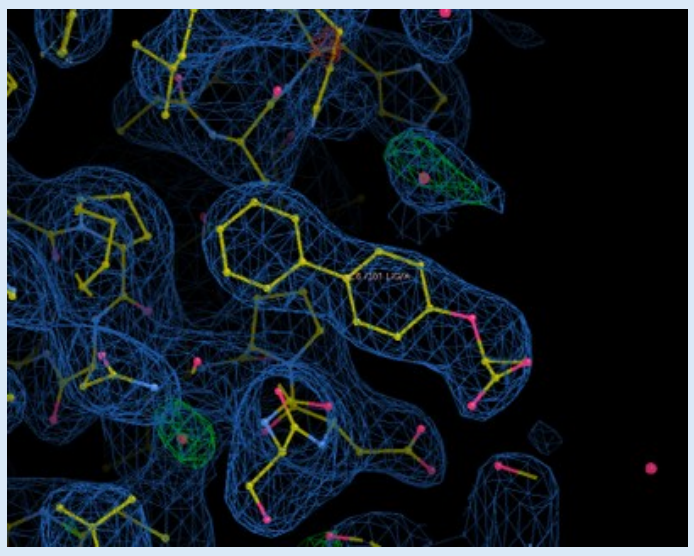
231		**Structure:** 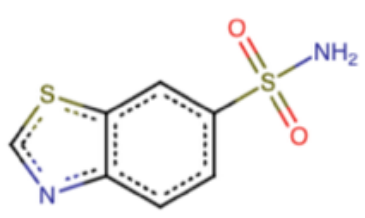 **SMILES string:** NS(=O)(=O)c1ccc2ncsc2c1 **Name:** Z1269184613	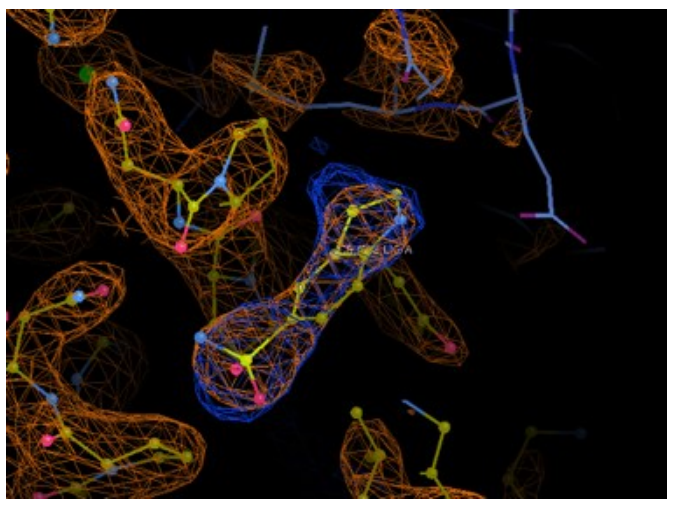	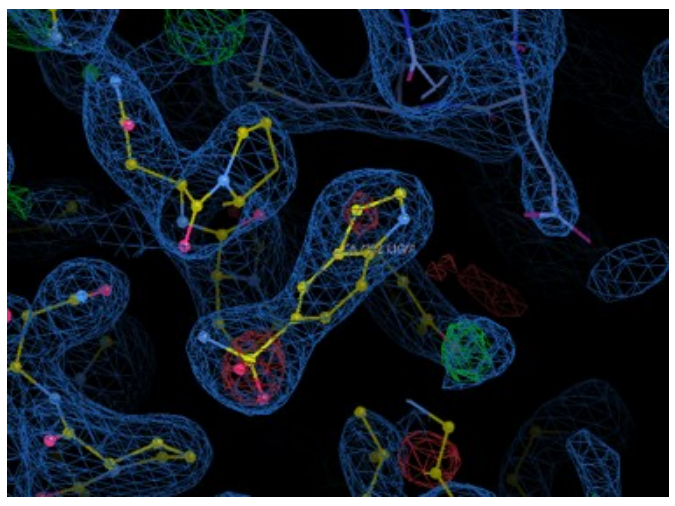
274		**Structure:** 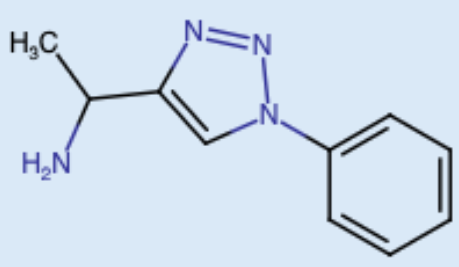 **SMILES string:** Cl.CC(N)c1cn(nn1)c2ccccc2 **Name:** Z1428159350	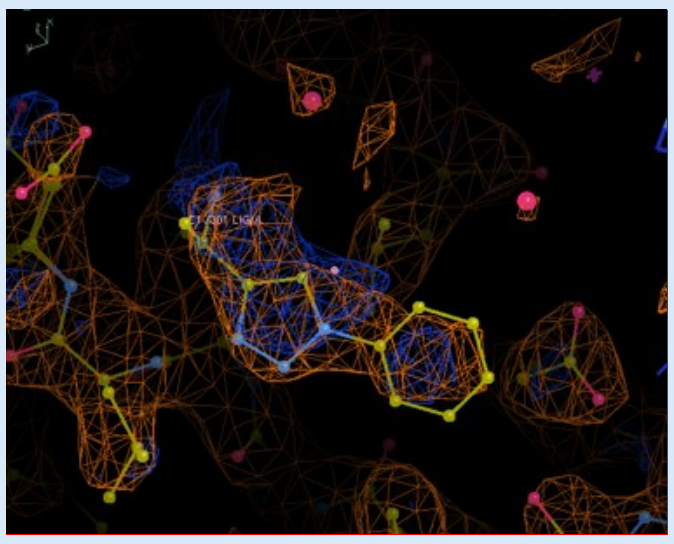	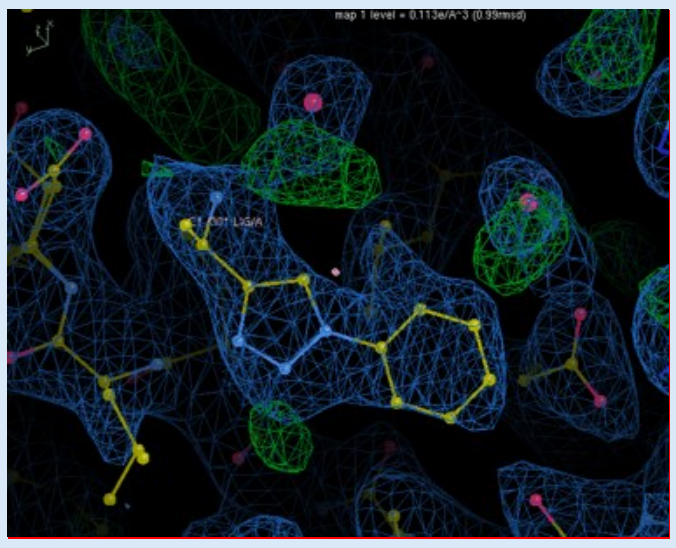

331		**Structure:**<br>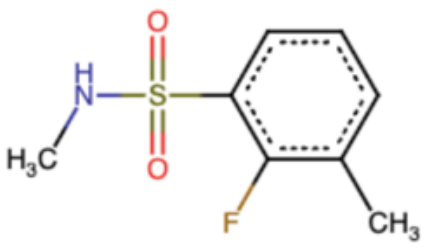<br>**SMILES string:**<br>CNS(=O)(=O)c1cccc(C)c1F<br>**Name:** Z1639162606	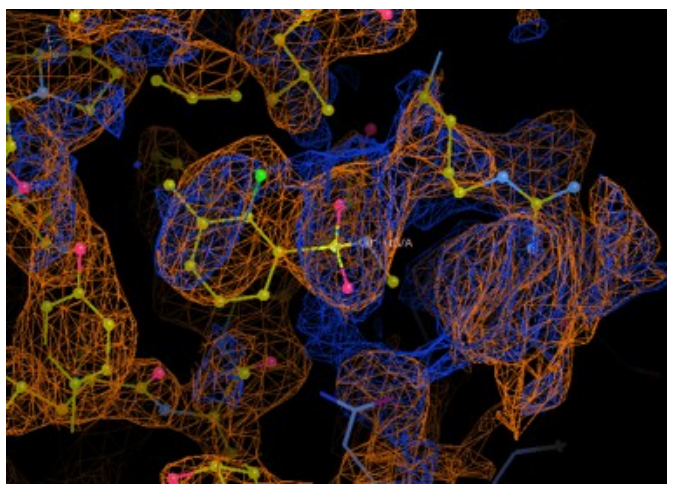	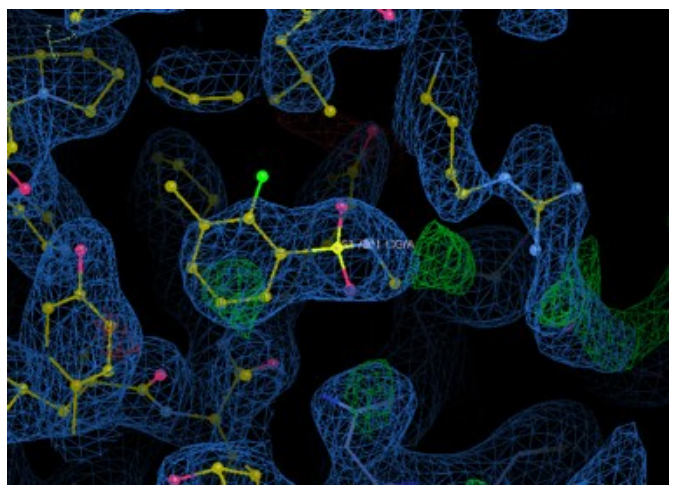
360		**Structure:**<br>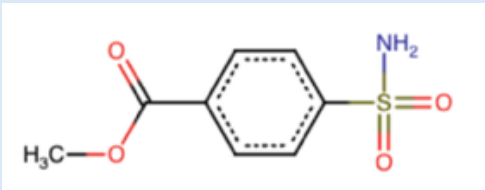<br>**SMILES string:**<br>COC(=O)c1ccc(cc1)S(N)(=O)=O<br>**Name:** Z18197050	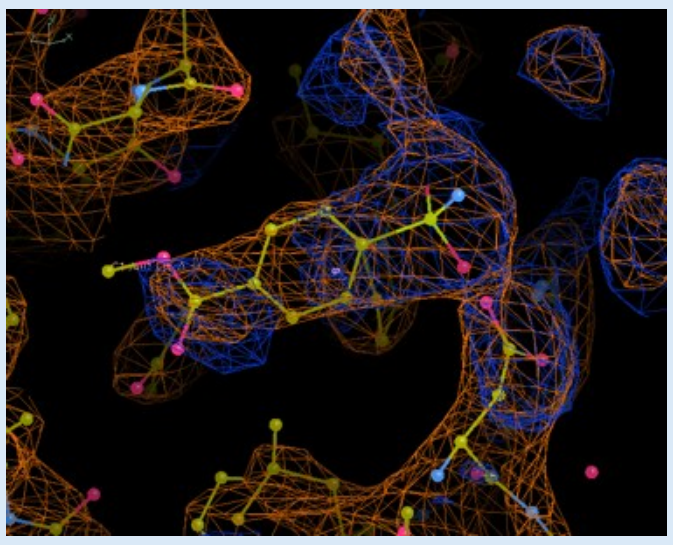	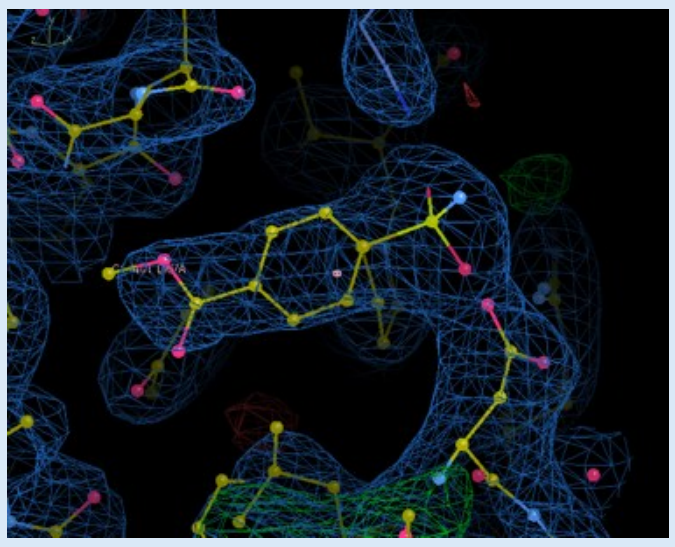
466		**Structure:**<br>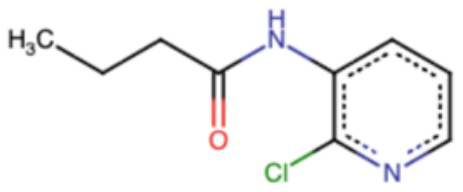<br>**SMILES string:**<br>CCCC(=O)Nc1cccnc1Cl<br>**Name:** Z240654968	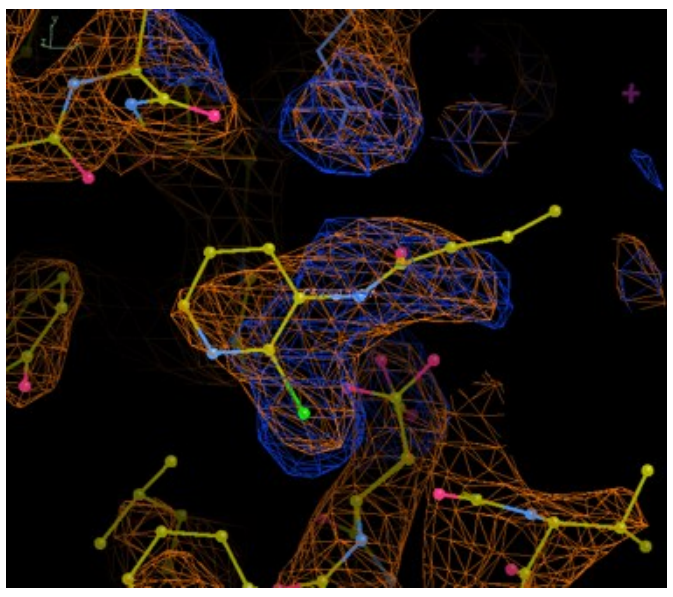	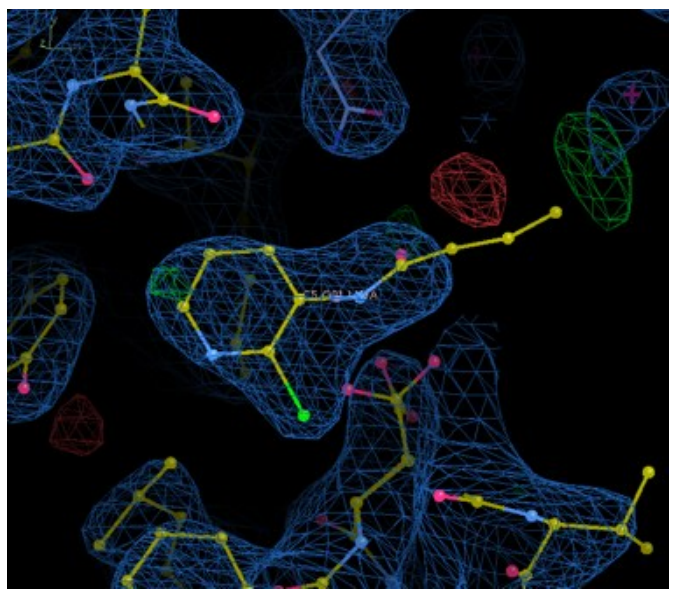
496		**Structure:**<br>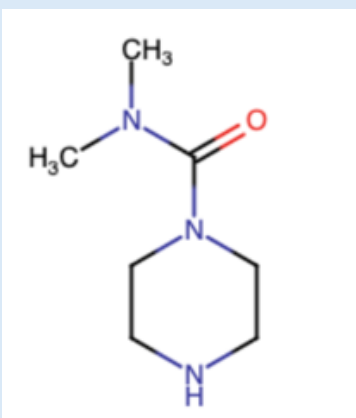<br>**SMILES string:**<br>CN(C)C(=O)N1CCNCC1<br>**Name:** Z270760338	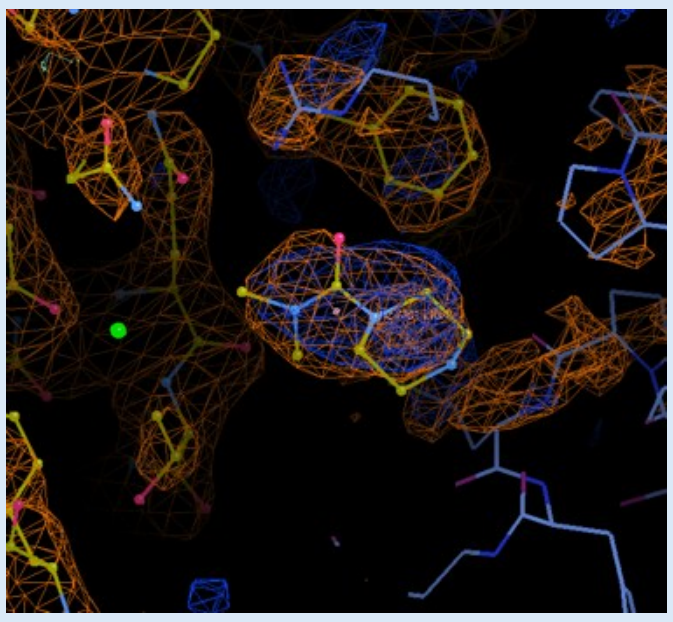	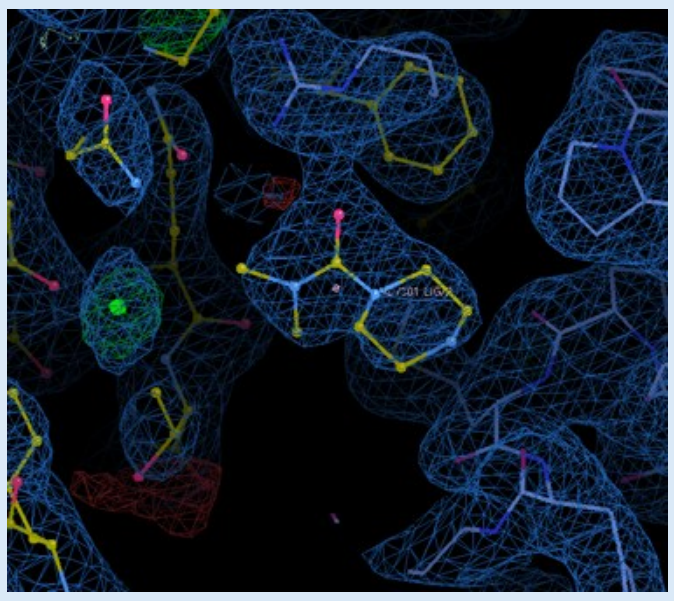
637		**Structure:**<br>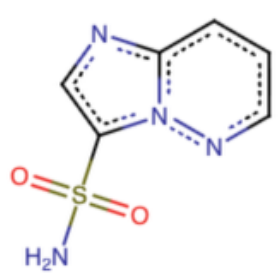		

		**SMILES string:** NS(=O)(=O)c1cnc2cccnn12<br><br>**Name:** Z3765495818	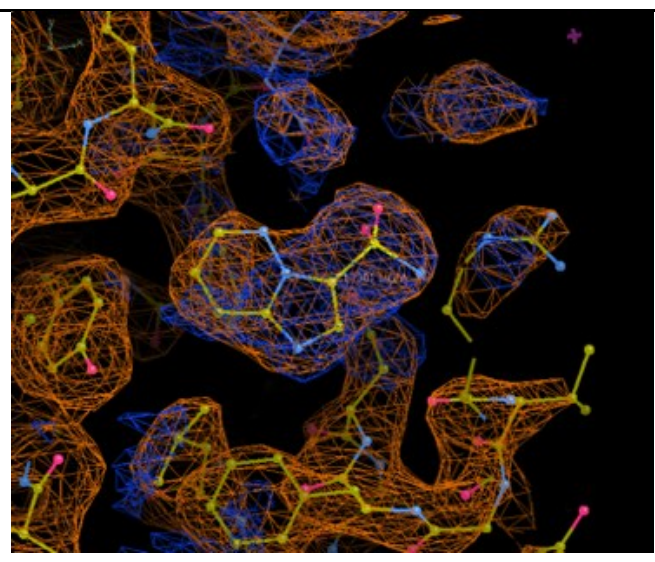	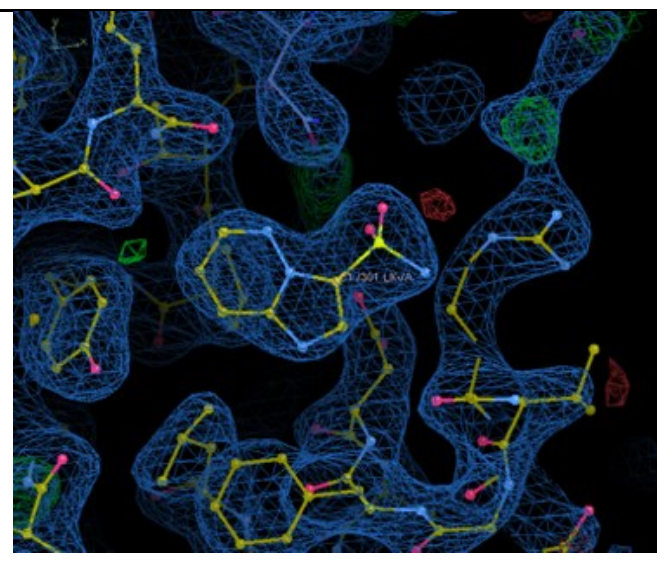
733		**Structure:**<br>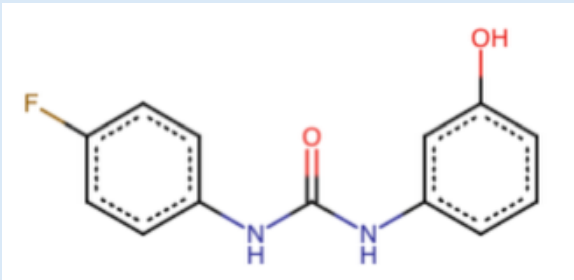<br><br>**SMILES string:** Oc1cccc(NC(=O)Nc2ccc(F) cc2)c1<br><br>**Name:** Z55290386	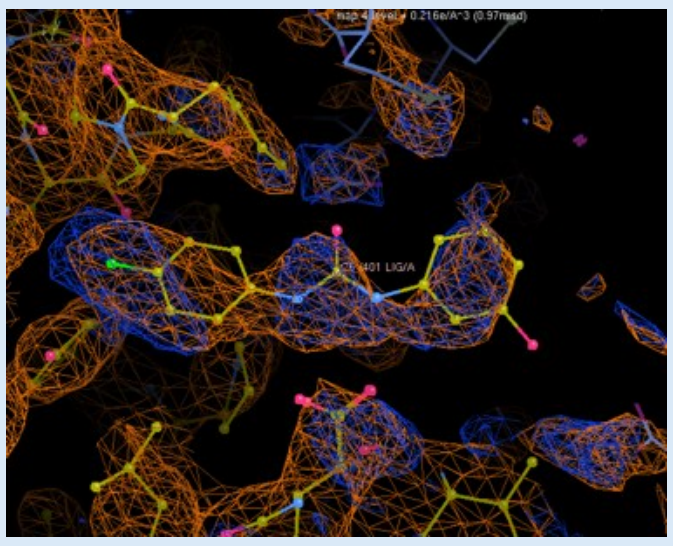	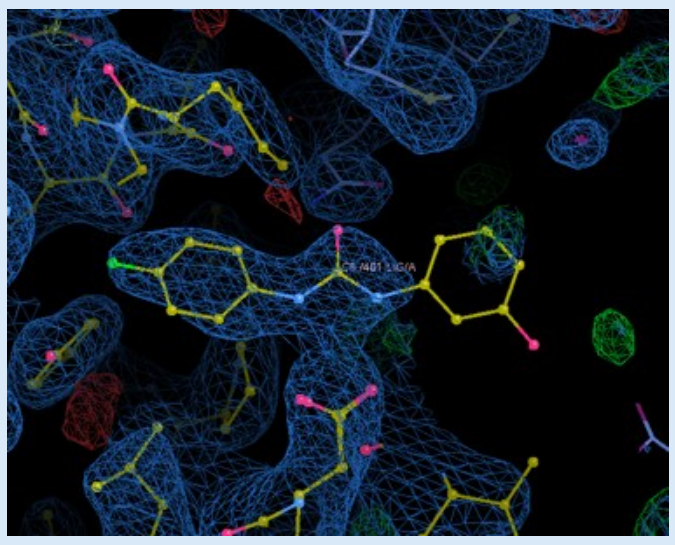

Table S5. **Physicochemical classification of amino acid residues interacting with each selected fragment.** Residues indicated with * interact with the fragment through a water bridge, while residues indicated with # are residues from the crystallographic symmetric of UbC4.

<table>
<tr><th rowspan="2">Cavity</th><th rowspan="2">Fragment</th><th colspan="5">Residues type</th></tr>
<tr><th>non-polar</th><th>polar</th><th>acidic</th><th>basic</th><th>aromatic</th></tr>
<tr><td rowspan="5">3</td><td>331</td><td>Pro122</td><td></td><td>His82
Asp144</td><td></td><td></td></tr>
<tr><td>360</td><td>Leu147</td><td></td><td>His82</td><td>Arg140</td><td>Tyr121</td></tr>
<tr><td>466</td><td></td><td></td><td></td><td>Arg140</td><td>Tyr121</td></tr>
<tr><td>637</td><td>Leu147</td><td></td><td>His82</td><td></td><td>Tyr121</td></tr>
<tr><td>733</td><td>Leu147
Leu136</td><td></td><td>Asp144</td><td></td><td>Phe143</td></tr>
<tr><td>15</td><td>274</td><td>Leu128
Leu94</td><td></td><td>Asp126*</td><td></td><td></td></tr>
<tr><td rowspan="3">2</td><td>96</td><td>Pro83
Leu136
Pro124
Met19#</td><td></td><td></td><td>Arg15#</td><td>Tyr121</td></tr>
<tr><td>116</td><td>Pro83
Pro122
A133</td><td></td><td></td><td>Arg15#</td><td></td></tr>
<tr><td>231</td><td></td><td>Asn123</td><td>Glu18#</td><td></td><td></td></tr>
<tr><td>14</td><td>496</td><td></td><td></td><td>Asp20#</td><td></td><td></td></tr>
</table>

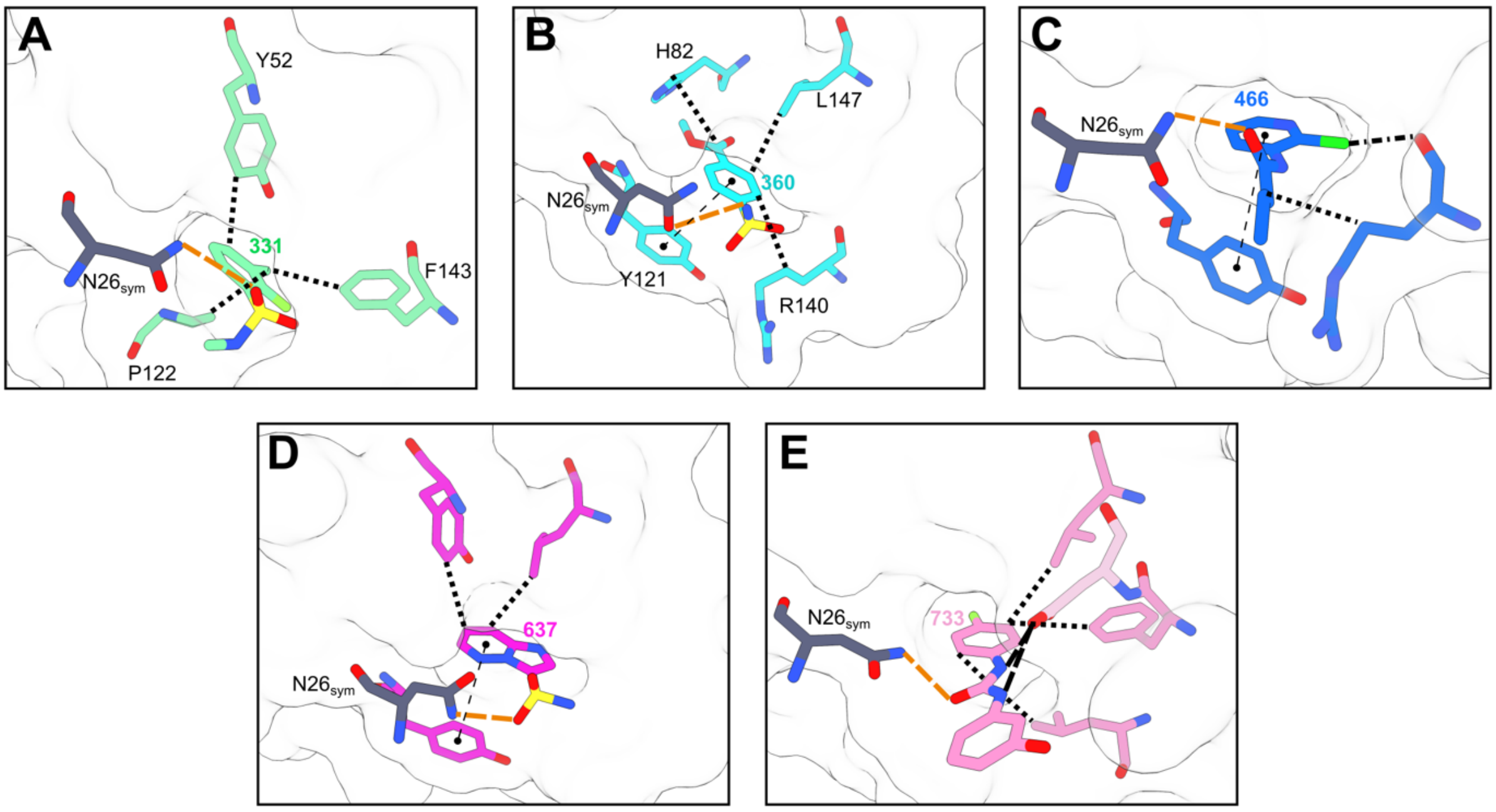

Figure S8. Asn26, from a crystallographic symmetric, forms a H-bond with fragments in cavity 3. The interactions between UbC4 and fragments 331 (A), 360 (B), 466 (C), 637 (D) and 733 (E) were identified using the Protein-Ligand Interaction Profiler (PLIP) webserver (https://plip-tool.biotec.tu-dresden.de/ (52)) and they are displayed as dotted lines for hydrophobic interactions, as dashed lines with endpoints for π-π interactions, as strong dashed lines for H-bonds and electrostatic interactions, and as mixed dashed-dotted lines for halogen bonds. Asn26 from the UbC4 crystallographic symmetric is shown in dark grey and interactions formed between these residues and fragments are shown in orange. Residues and fragments are represented as sticks.

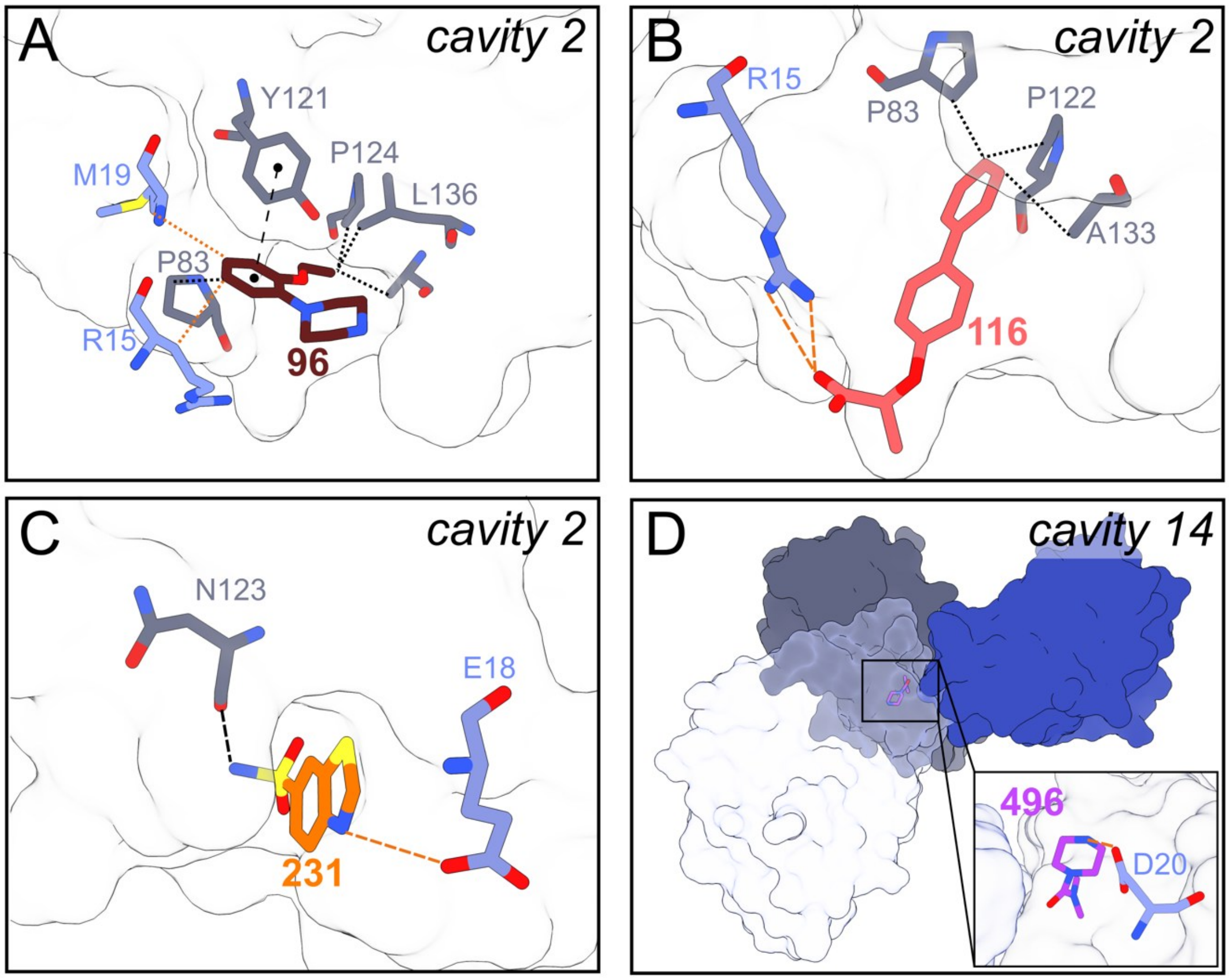

Figure S9. Fragments 96, 116, 231 and 496 are stabilized by crystal contacts. The interactions between UbC4 and fragments 96 (A), 116 (B), 231 (C), 496 (D and insert) were identified using the Protein-Ligand Interaction Profiler (PLIP) webserver (https://plip-tool.biotec.tu-dresden.de/ (52)) and they are displayed as dotted lines for hydrophobic interactions, as dashed lines with endpoints for π-π interactions, as strong dashed lines for H-bonds and electrostatic interactions, and as mixed dashed-dotted lines for halogen bonds. Residues from the UbC4 crystallographic symmetric are shown in light blue and interactions formed between these residues and fragments are shown in orange. Residues and fragments are represented as sticks.

Table S6. In silico ADMET analysis. The ADMET prediction has been carried out for the original fragments 274, 331, 360, 637, 733 and their corresponding elongated fragments using the https://www.swissadme.ch/ webtool. Abbreviations: ADMET - absorption, distribution, metabolism, excretion, and toxicity; TPSA - topological polar surface area; GI absorption - gastrointestinal absorption; BBB permeability - blood brain barrier permeability; PgP substrate - P-glycoprotein substrate. These predictions provide a preliminary assessment of the drug-like character of the proposed compounds.

Fragment	274	CE011	CE012	CE021	CE022
Formula	C10H12N4	C10H12N4O	C11H14N4O	C12H17N4+	C12H14N4O
MW (g/mol)	188.23	204.23	218.26	217.29	230.27
SMILES	CC(N)c1cn(nn1)c2cccc c2	C[C@@](N)(c1cn(nn1)c1ccccc 1)O	C[C@@](N)(c1cn(nn1)c1ccccc1) OC	CC[NH2+]C(c1nnn(c1)c1ccccc 1)C	C[C@@H](NC(=O)C)c1cn(nn1)c1ccc cc1
Structure	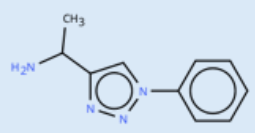	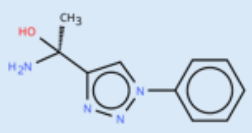	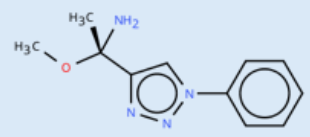	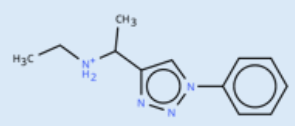	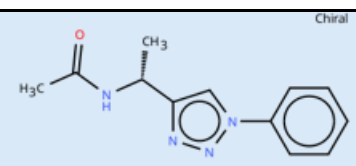
IUPAC name	1-(1-phenyltriazol-4-yl)ethanamine				
Consensus logP	1.10	0.55	1.07	0.88	1.33
Hbond donors	1	2	1	1	1
Hbond acceptors	3	4	4	2	3
Rotable bonds	2	2	3	4	4
Fraction sp3	0.20	0.20	0.27	0.33	0.25
TPSA (Angstrom 2)	56.73	76.96	59.61	47.32	63.75
logS (ESOL)	-1.86	-1.44	-1.76	-3.9	-2.05
GI absorption	High	High	High	High	High
BBB permeability	Yes	No	No	Yes	Yes
PgP substrate	No	No	No	No	No
Lipinski violations	0	0	0	0	0

Fragment	CE023	CE024	CE025	CE026	CE027
Formula	C11H14N4	C12H14N4O	C13H16N4O	C13H18N4	C13H16N4O
MW (g/mol)	202.26	230.27	244.29	230.31	244.29
SMILES	C[C@@H](NC)c1cn(nn1)c1 ccccc1	C[C@@H](NCC=O)c1cn(nn1)c 1ccccc1	C[C@@H](NCCC=O)c1cn(nn1)c 1ccccc1	C[C@@H](NCCC)c1cn(nn1)c 1ccccc1	C[C@@H](NC(=O)CC)c1cn(nn1) c1ccccc1
Structure	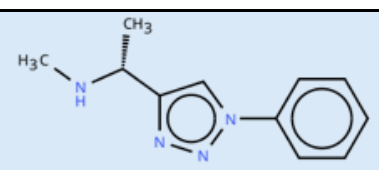	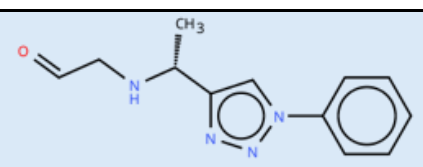	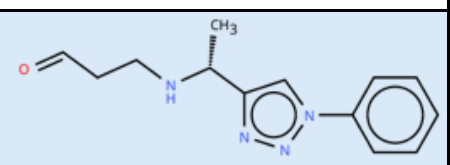	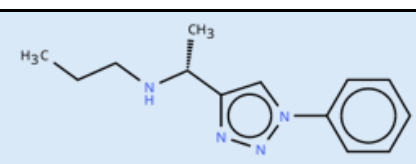	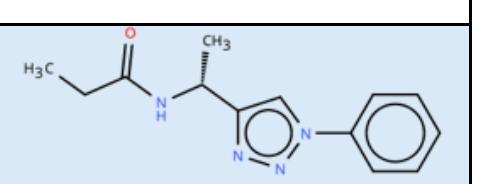
IUPAC name					

**clogP**	1.48	1.09	1.30	2.14	1.60
**Hbond donors**	1	1	1	1	1
**Hbond acceptors**	3	4	4	3	3
**Rotable bonds**	3	5	6	5	5
**Fraction sp3**	0.27	0.25	0.31	0.38	0.31
**TPSA (Angstrom 2)**	58.74	59.81	59.81	42.74	59.81
**logS (ESOL)**	-2.16	-1.84	-1.78	-2.71	-2.34
**GI absorption**	High	High	High	High	High
**BBB permeability**	Yes	Yes	Yes	Yes	Yes
**PgP substrate**	No	No	No	No	No
**Lipinski violations**	0	0	0	0	0

**Fragment**	**331**	**CE031**	**CE032**	**CE033**	**CE034**
**Formula**	C8H10FNO2S	C8H10FNO3S	C9H12FNO3S	C8H10FNO3S	C8H10FNO3S
**MW (g/mol)**	203.23	219.23	233.26	219.23	219.23
**SMILES**	CNS(=O)(=O)c1cccc(C)c1F	CNS(=O)(=O)c1ccc(c(C)c1F)O	CNS(=O)(=O)c1ccc(c(C)c1F)OC	CNS(=O)(=O)c1cccc(CO)c1F	CNS(=O)(=O)c1cc(cc(C)c1F)O
**Structure**	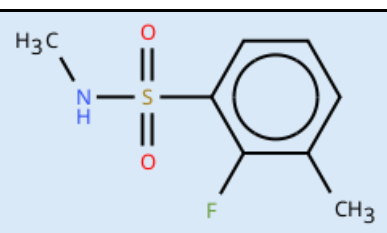	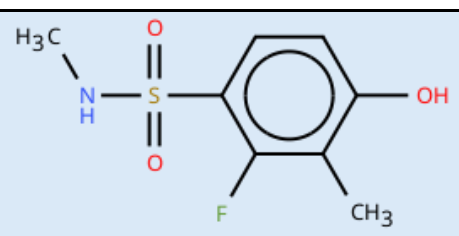	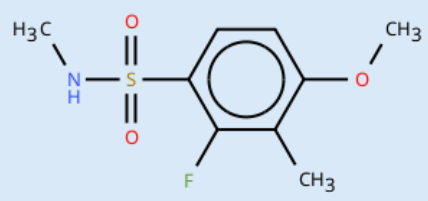	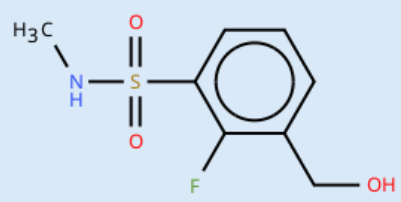	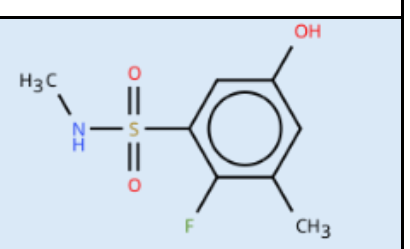
**IUPAC name**	2-fluoro-N,3-dimethylbenzenesulfonamide	4-hydroxy-2-fluoro-N,3-dimethylbenzenesulfonamide	2-fluoro-4-methoxy-N,3-dimethylbenzenesulfonamide	2-fluoro-3-hydroxymethyl-N-methylbenzenesulfonamide	2-fluoro-5-hydroxy-N,3-dimethylbenzenesulfonamide
**clogP**	1.58	1.22	1.58	0.78	1.20
**Hbond donors**	1	2	1	2	2
**Hbond acceptors**	4	5	5	5	5
**Rotable bonds**	2	2	3	3	2
**Fraction sp3**	0.25	0.25	0.33	0.25	0.25
**TPSA (Angstrom 2)**	54.55	74.78	63.78	74.78	74.78
**logS (ESOL)**	-2.16	-2.01	-2.22	-1.38	-2.01
**GI absorption**	High	High	High	High	High
**BBB permeability**	Yes	Yes	Yes	No	Yes
**PgP substrate**	No	No	No	No	No

**Lipinski violations**	0	0	0	0	0

**Fragment**	**360**	**CE041**	**CE042**	**CE043**
**Formula**	C8H9NO4S	C9H11NO4S	C9H12NO4S	C9H13NO4S
**MW (g/mol)**	215.23	229.25	230.26	230.26
**SMILES**	COC(=O)c1ccc(cc1)S(=O)(=O)N	C(OC(=O)c1ccc(cc1)S(=O)(=O)N)C	CO/C(=[O]\C)/c1ccc(cc1)S(=O)(=O)N	CO[C](=O)(c1ccc(cc1)S(=O)(=O)N)C
**Structure**	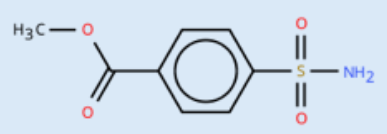	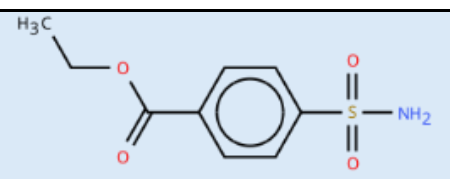	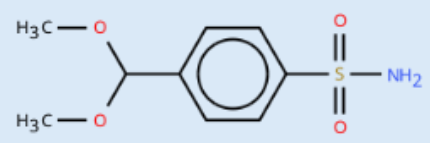	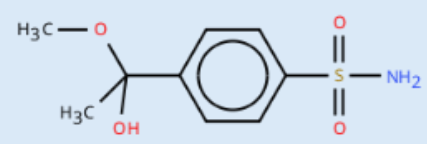
**IUPAC name**	methyl 4-sulfamoylbenzoate	ethyl 4-sulfamoylbenzoate		
**clogP**	0.66	0.88	-0.14	-0.41
**Hbond donors**	1	1	1	1
**Hbond acceptors**	5	5	5	5
**Rotable bonds**	3	4	3	3
**Fraction sp3**	0.12	0.22	0.22	0.22
**TPSA (Angstrom 2)**	94.84	94.84	94.84	94.84
**logS (ESOL)**	-1.70	-2.03	-1.72	-1.51
**GI absorption**	High	High	High	High
**BBB permeability**	No	No	No	No
**PgP substrate**	No	No	Yes	Yes
**Lipinski violations**	0	0	0	0

**Fragment**	**466**	**CE051**	**CE052**	**CE053**	**CE054**	**CE055**	**CE056**
**Formula**	C9H11ClN2O	C10H13ClN2O	C9H11ClN2O2	C10H13ClN2O2	C10H14ClN2O	C11H16ClN2O	C10H14ClN2O2
**MW (g/mol)**	198.65	212.68	214.65	228.68	213.68	227.71	229.68
**SMILES**	CCCC(=O)N c1cccnc1Cl	CCCC(=O)Nc 1ccc(nc1Cl)C	CCCC(=O)Nc1cc(cnc1Cl)O	CCCC(=O)Nc1cc(cnc1Cl)OC	CCCC(=O)NC1=CC=C[N](=C1Cl)C	CCCC(=O)NC1=CC=C[N](=C1Cl)CC	CCCC(=O)NC1=CC=C[N](=C1Cl)CO
**Structure**	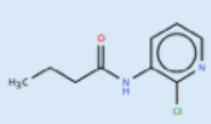	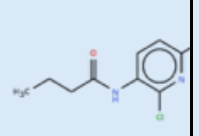	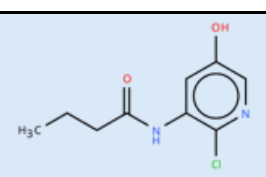	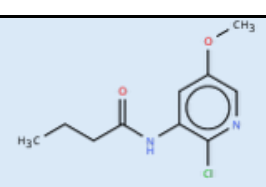	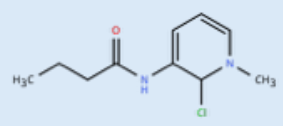	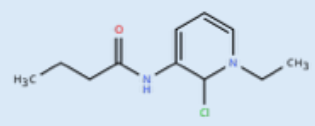	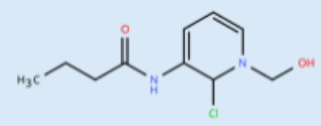
**IUPAC name**	N-(2-chloro-3-pyridinyl)butanamide						
**clogP**	1.92	2.27	1.52	1.94	0.91	1.28	0.36
**Hbond donor33s**	1	1	2	1	1	1	2
**H54bond acceptors**	2	2	3	3	1	1	2
**Rotable bonds**	4	4	4	5	4	5	5

**Fraction sp3**	0.33	0.40	0.33	0.40	0.40	0.45	0.40
**TPSA (Angstrom 2)**	41.99	41.99	62.22	51.22	34.03	34.03	54.26
**logS (ESOL)**	-2.30	-2.61	-2.14	-2.35	-2.45	-2.64	-2.05
**GI absorption**	High	High	High	High	High	High	High
**BBB permeability**	Yes	Yes	Yes	Yes	Yes	Yes	Yes
**PgP substrate**	No	No	No	No	No	No	No
**Lipinski violations**	0	0	0	0	0	0	0

**Fragment**	**637**	**Ce061**	**CE062**	**CE063**
**Formula**	C6H6N4O2S	C7H8N4O2S	C7H8N4O3S	C7H6N4O3S
**MW (g/mol)**	198.20	212.23	228.23	226.21
**SMILES**	NS(=O)(=O)c1cnc2cccnn12	NS(=O)(=O)c1cnc2cc(cnn12)C	NS(=O)(=O)c1cnc2cc(cnn12)CO	NS(=O)(=O)c1cnc2cc(cnn12)C=O
**Structure**	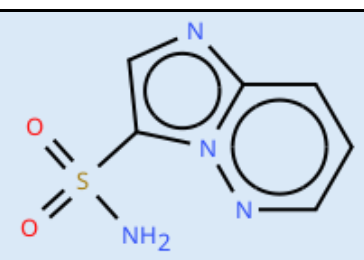	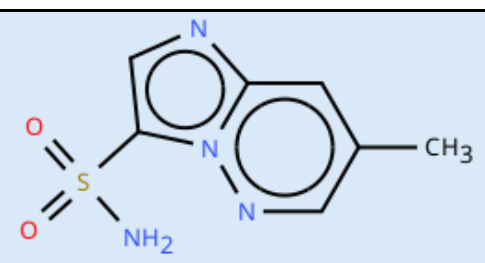	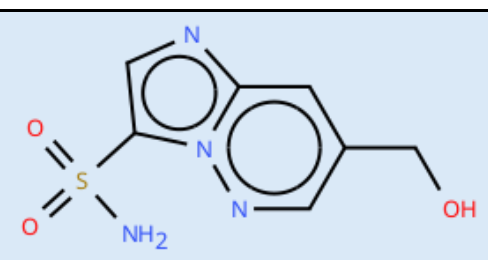	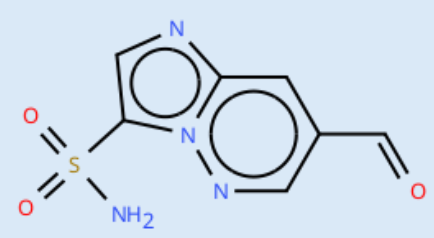
**IUPAC name**	imidazo[1,2-b]pyridazine-3-sulfonamide			
**clogP**	-0.54	-0.10	-0.98	-0.81
**Hbond donors**	1	1	2	1
**Hbond acceptors**	5	5	6	6
**Rotable bonds**	1	1	2	2
**Fraction sp3**	0.00	0.14	0.14	-0.81
**TPSA (Angstrom 2)**	98.73	98.73	118.96	115.80
**logS (ESOL)**	-1.02	-1.31	-0.52	-0.73
**GI absorption**	High	High	High	High
**BBB permeability**	No	No	No	No
**PgP substrate**	No	No	No	No
**Lipinski violations**	0	0	0	0

**Fragment**	**CE064**	**CE065**	**CE066**	**CE067**
**Formula**	C7H10N5O2S	C6H7N5O2S	C7H9N5O2S	C7H8N4O2S
**MW (g/mol)**	228.25	213.22	227.24	212.23
**SMILES**	NS(=O)(=O)c1cnc2cc(cnn12)C[NH3]	NS(=O)(=O)c1cnc2ccc(nn12)N	NS(=O)(=O)c1cnc2ccc(nn12)NC	NS(=O)(=O)c1cnc2c(ccnn12)C

**Structure**	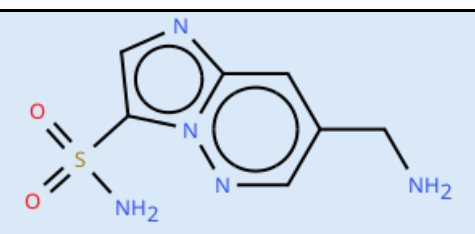	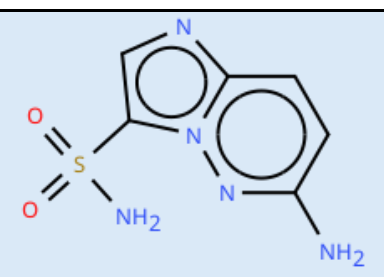	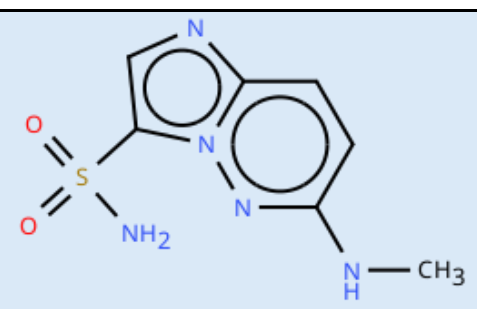	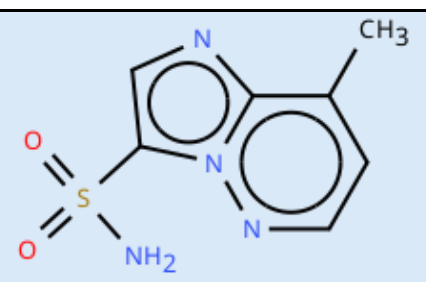
**IUPAC name**				
**clogP**	-2.31	-0.91	-0.50	-0.07
**Hbond donors**	2	2	2	1
**Hbond acceptors**	5	5	5	5
**Rotable bonds**	2	1	2	1
**Fraction sp3**	0.14	-0.87	0.14	0.14
**TPSA (Angstrom 2)**	101.97	124.75	110.76	98.73
**logS (ESOL)**	-0.36	-0.91	-1.27	-1.31
**GI absorption**	High	High	High	High
**BBB permeability**	No	No	No	No
**PgP substrate**	No	No	No	No
**Lipinski violations**	0	0	0	0

**Fragment**	**733**	**CE073**	**CE074**	**CE075**
**Formula**	C13H11FN2O2	C13H11ClFN2O2	C13H11FN2O3	C13H10ClFN2O2
**MW (g/mol)**	246.24	281.69	262.24	280.68
**SMILES**	Oc1cccc(NC(=O)Nc2ccc(F)cc2)c1	Oc1cc(NC(NC2C=CC(Cl)(F)CC=2)=O)ccc1	Oc1cccc(NC(=O)Nc2ccc(F)c(c2)O)c1	Oc1cccc(NC(=O)Nc2cc(c(F)cc2)Cl)c1
**Structure**	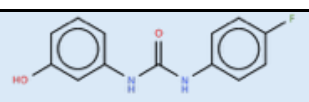	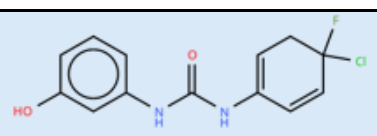	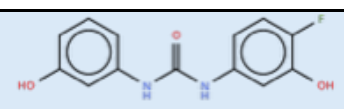	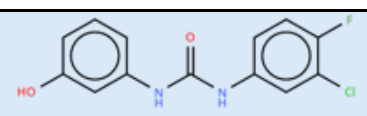
**IUPAC name**	1-(4-fluorophenyl)-3-(3-hydroxyphenyl)urea			1-(3-chloro-4-fluorophenyl)-3-(3-hydroxyphenyl)urea
**clogP**	2.38	1.90	1.99	3.10
**Hbond donors**	3	3	4	3
**Hbond acceptors**	3	3	4	3
**Rotable bonds**	4	4	4	4
**Fraction sp3**	0.00	0.00	0.00	0.00
**TPSA (Angstrom 2)**	61.36	61.36	81.59	61.36
**logS (ESOL)**	-2.99	-3.21	-2.85	-4.19
**GI absorption**	High	High	High	High
**BBB permeability**	Yes	Yes	No	Yes
**PgP substrate**	No	No	No	No
**Lipinski violations**	0	0	0	0

References

1. Olsen SK, Lima CD. Structure of a Ubiquitin E1-E2 Complex: Insights to E1-E2 Thioester Transfer. Mol Cell. 2013 Mar;49(5):884–96. doi:10.1016/j.molcel.2013.01.013

2. Middleton AJ, Day CL. The molecular basis of lysine 48 ubiquitin chain synthesis by Ube2K. Sci Rep. 2015 Nov 23;5(1):16793. doi:10.1038/srep16793
